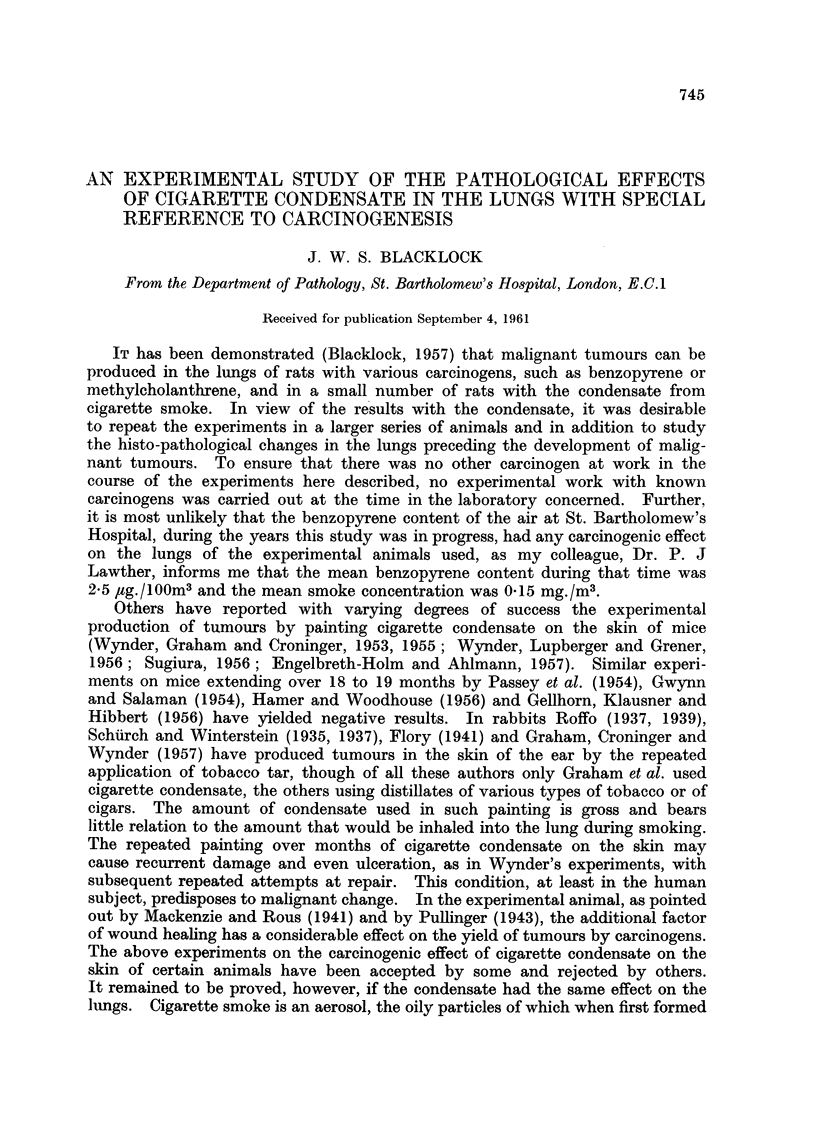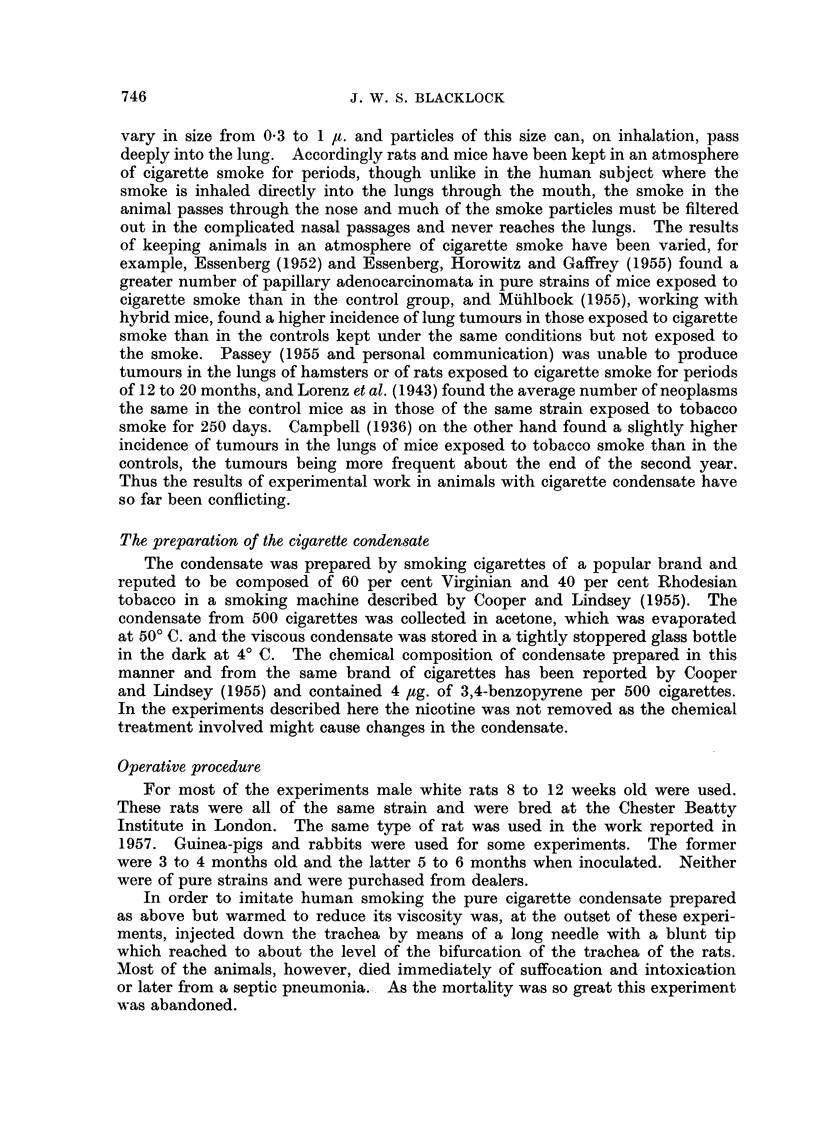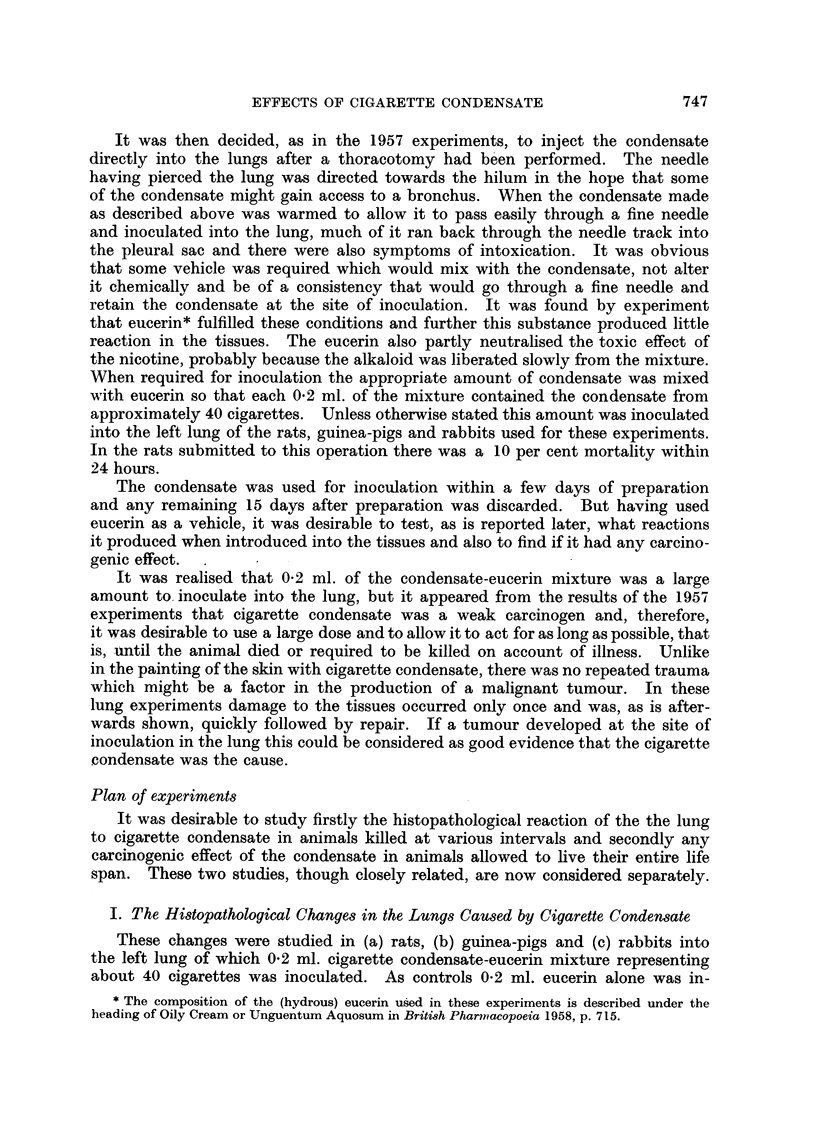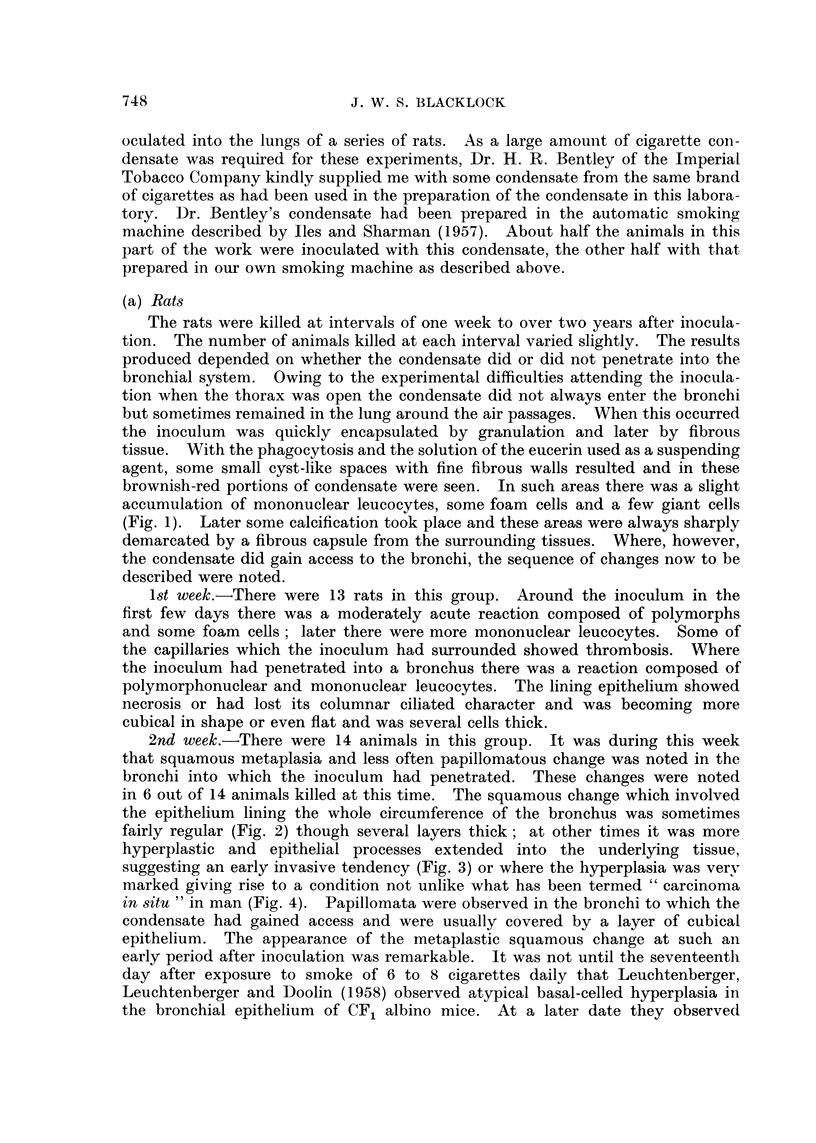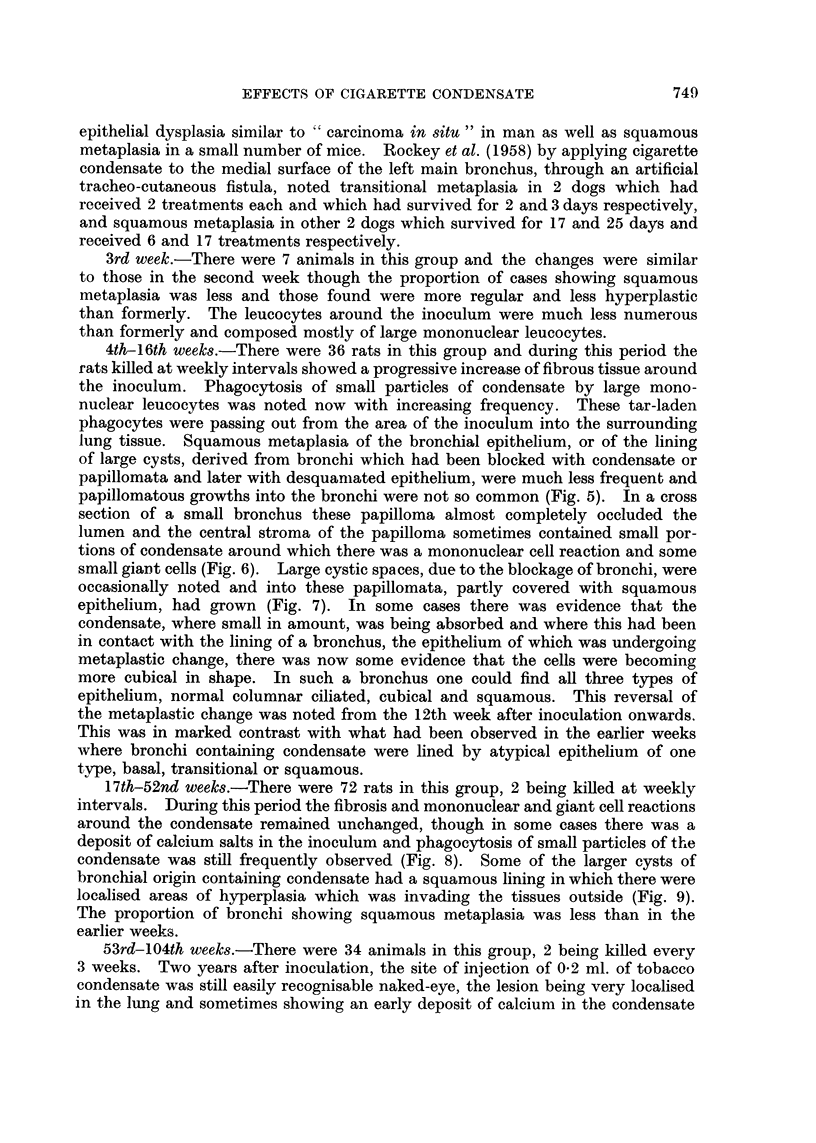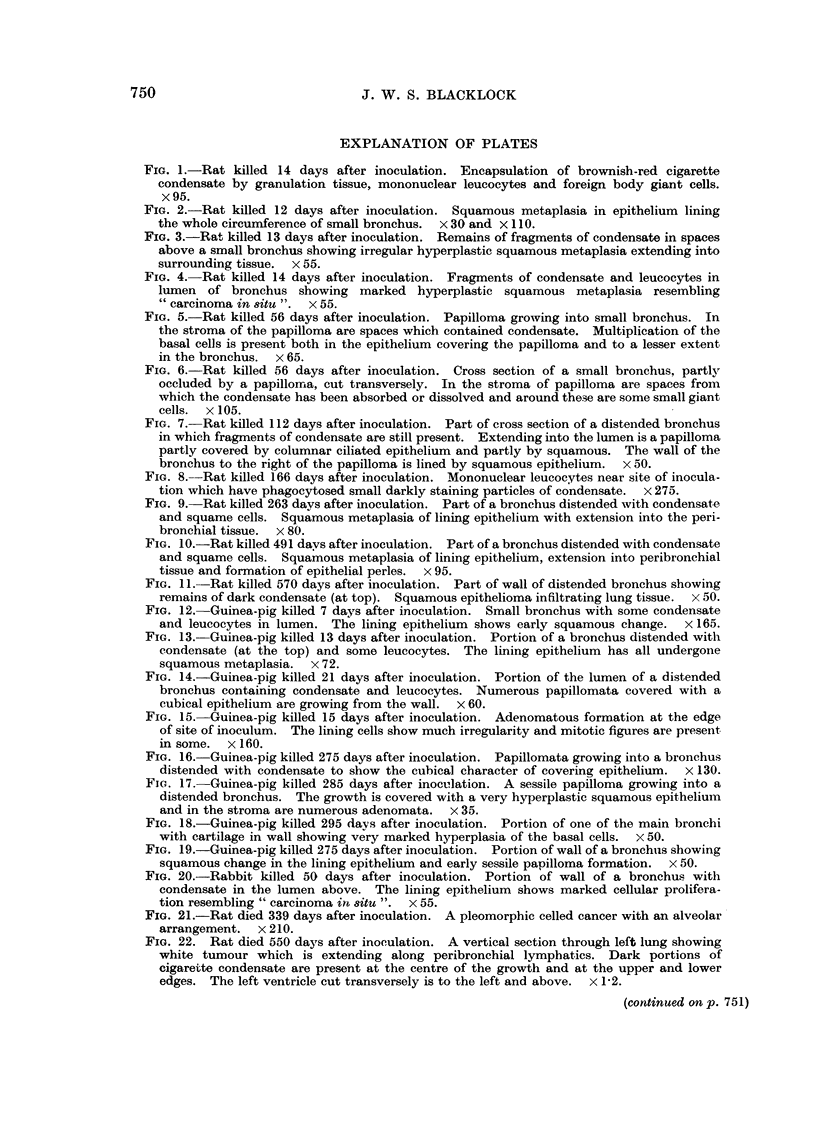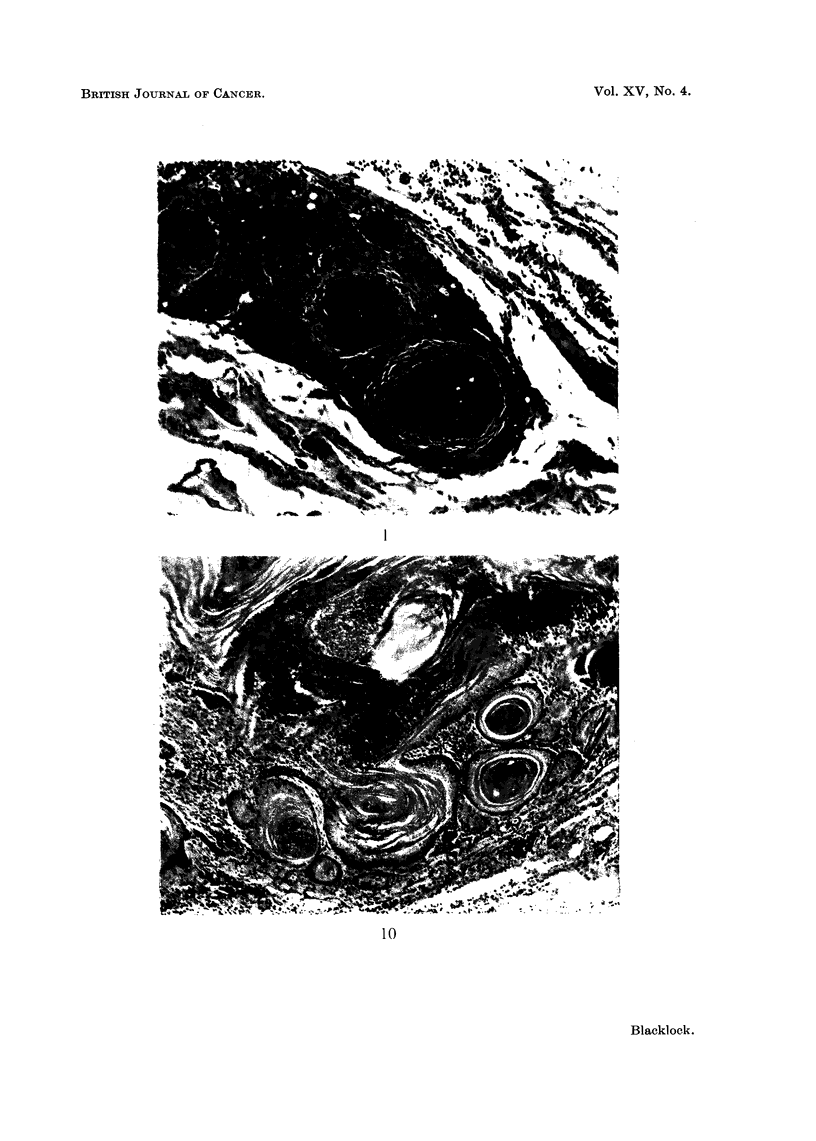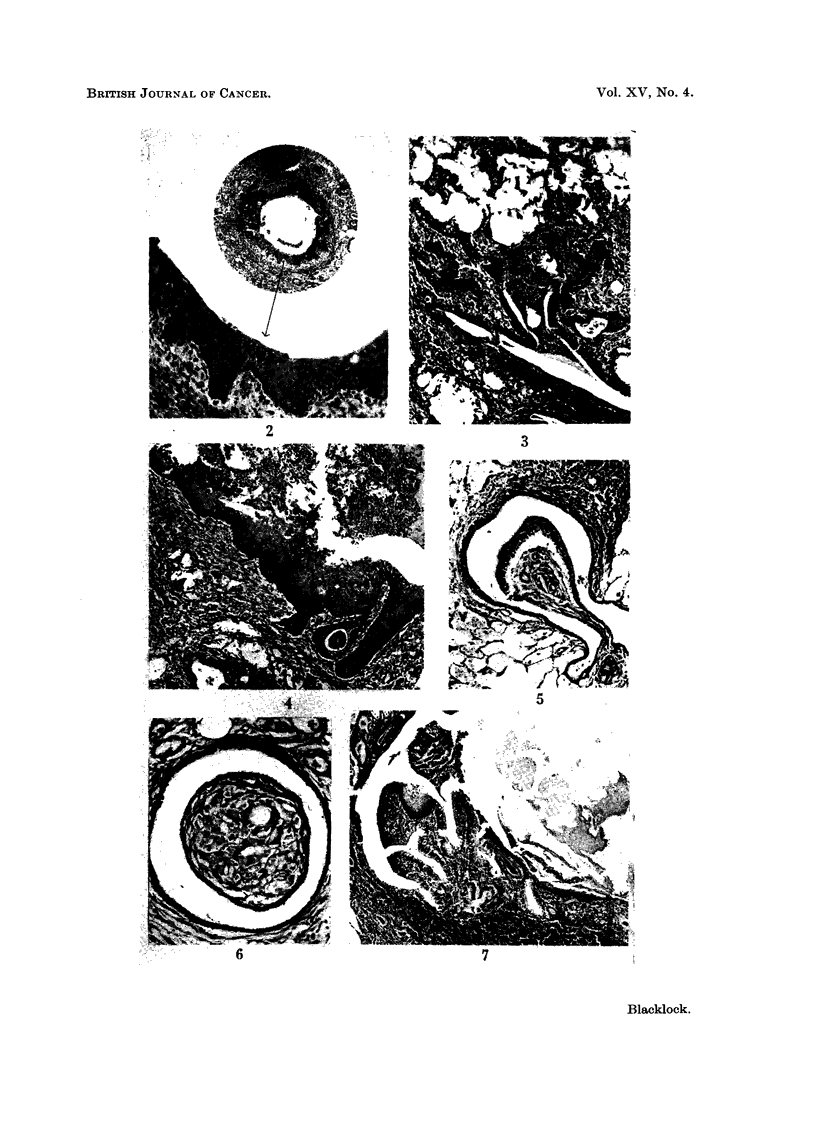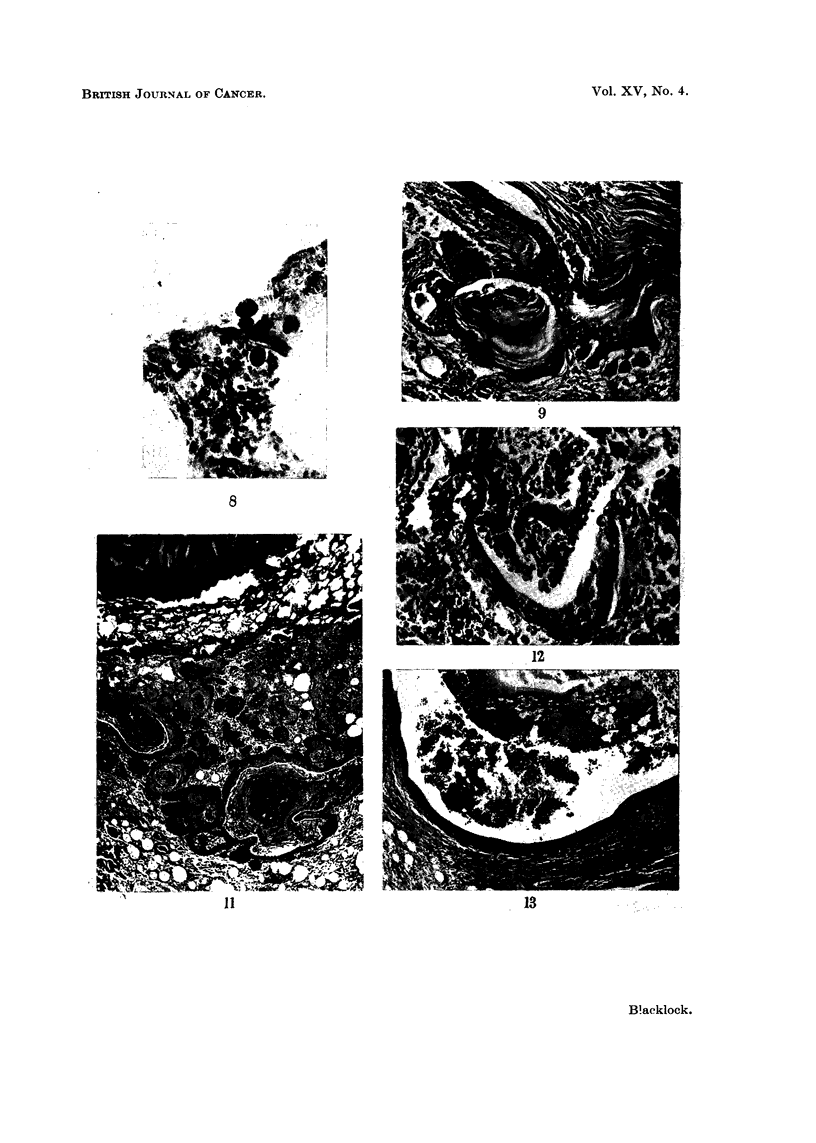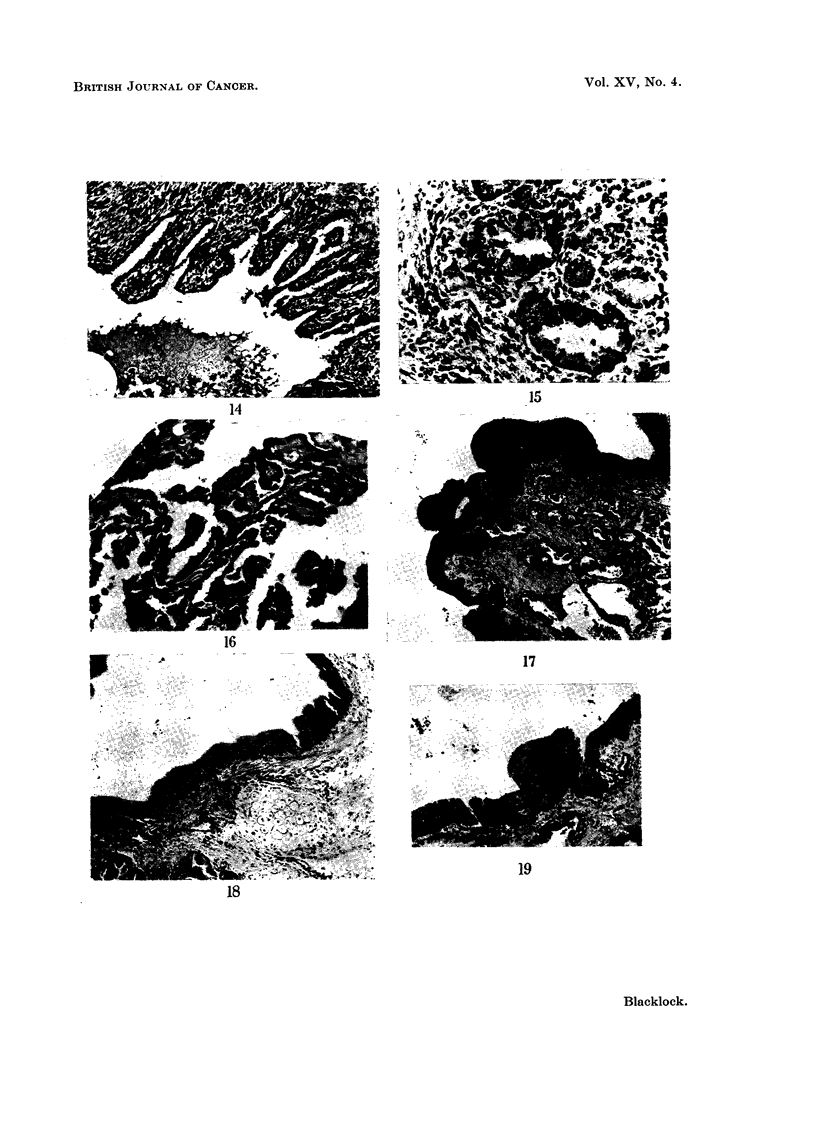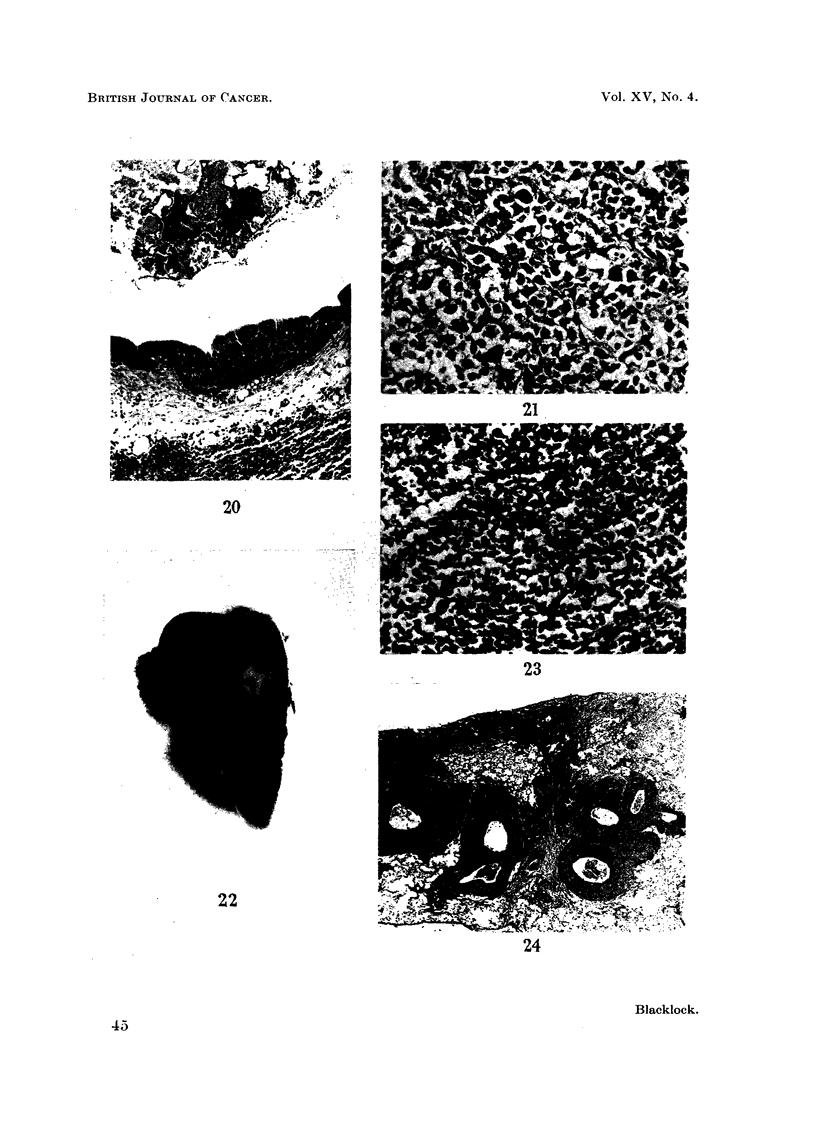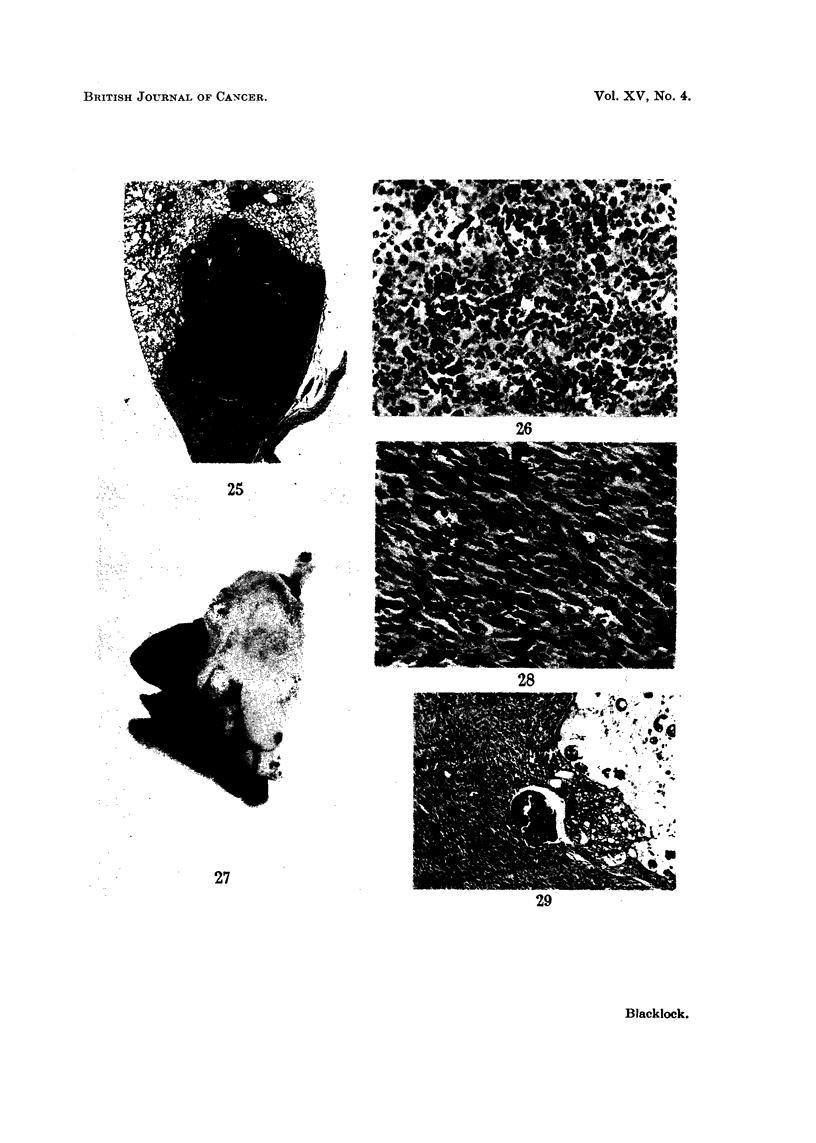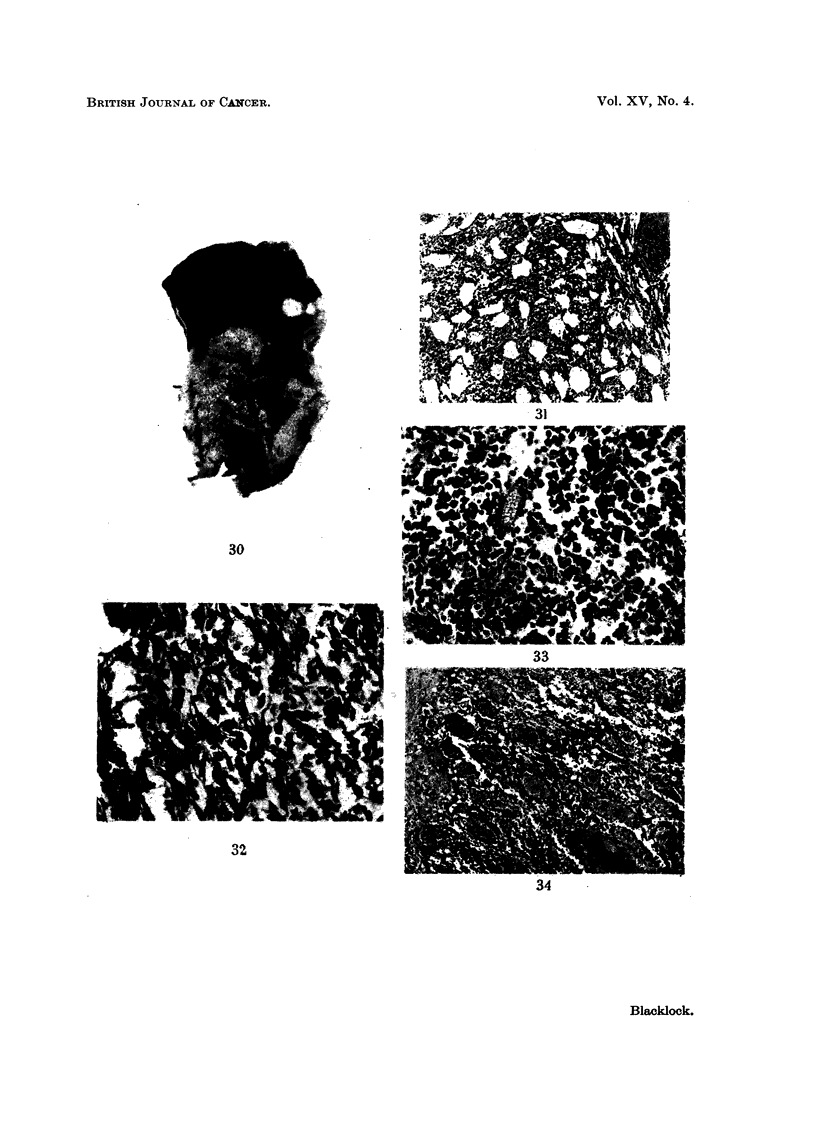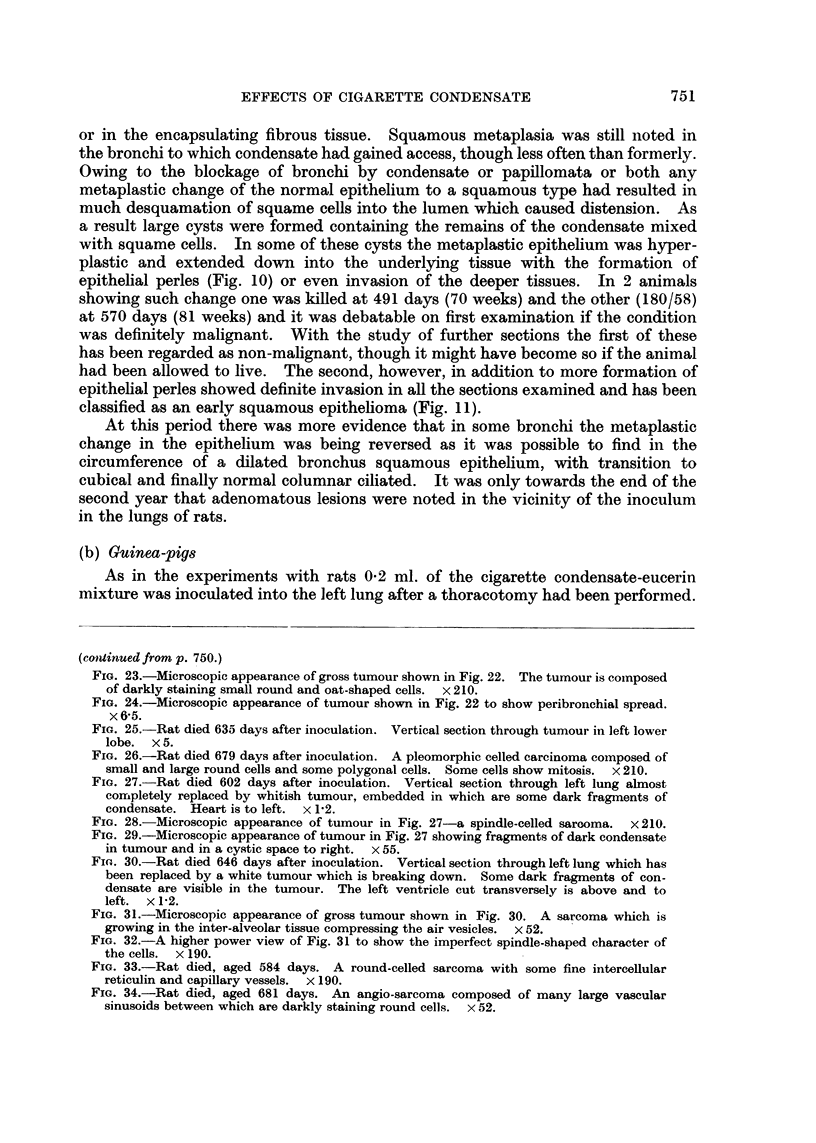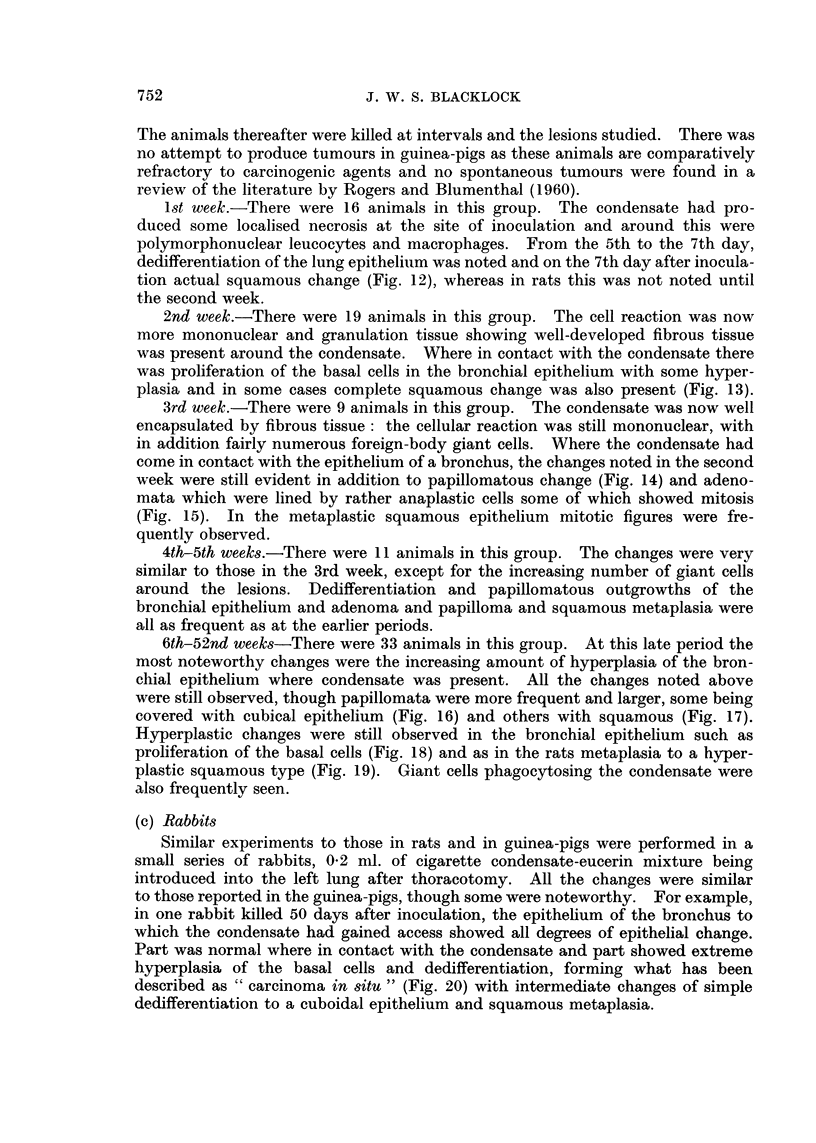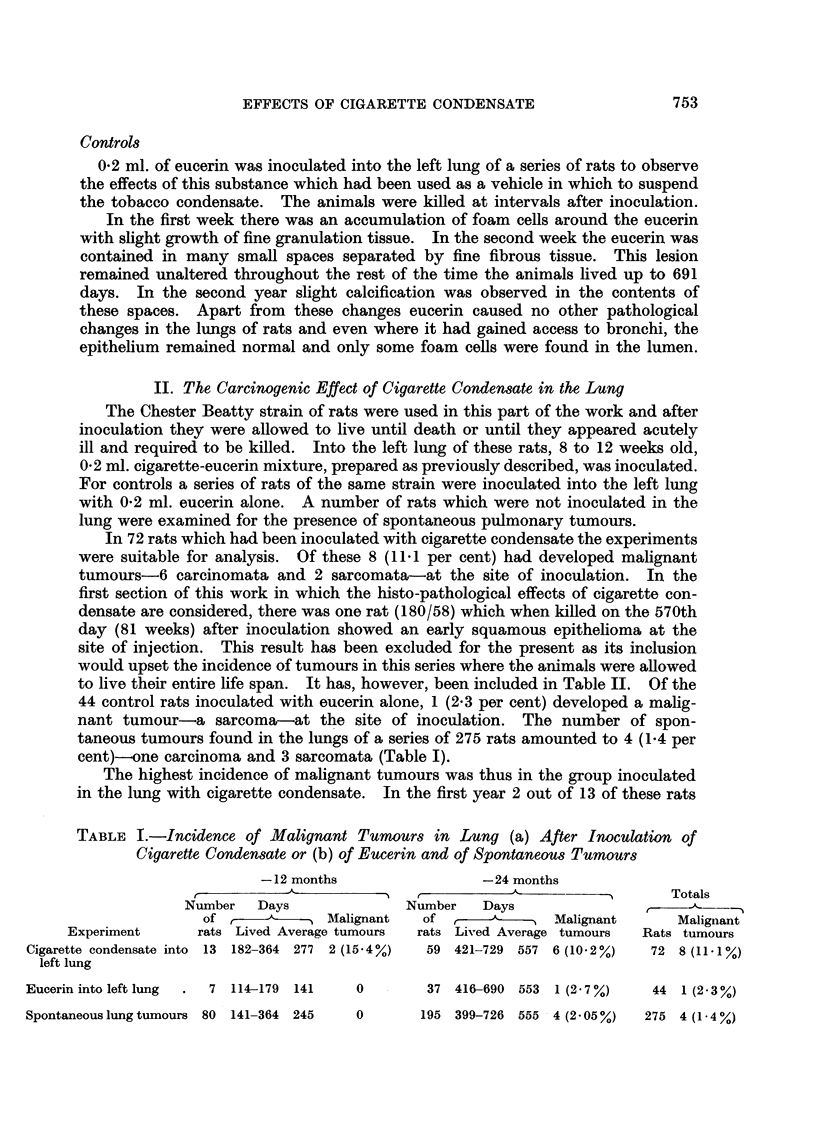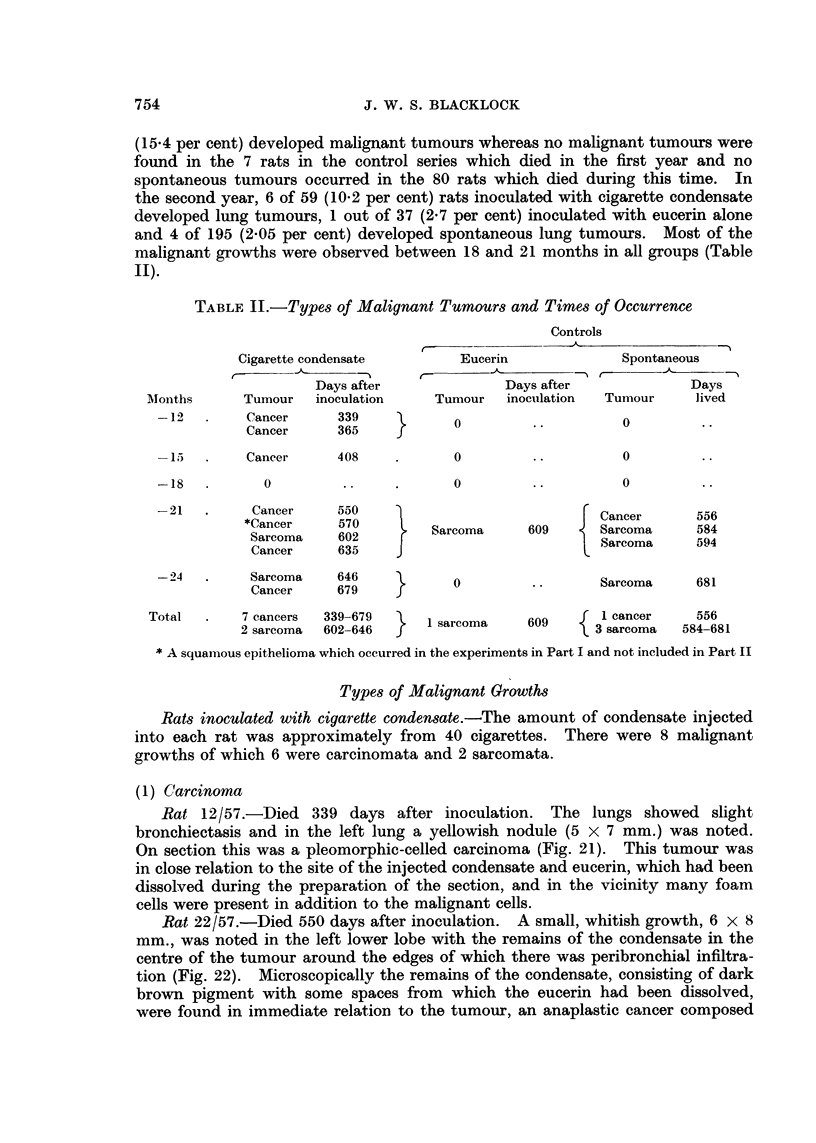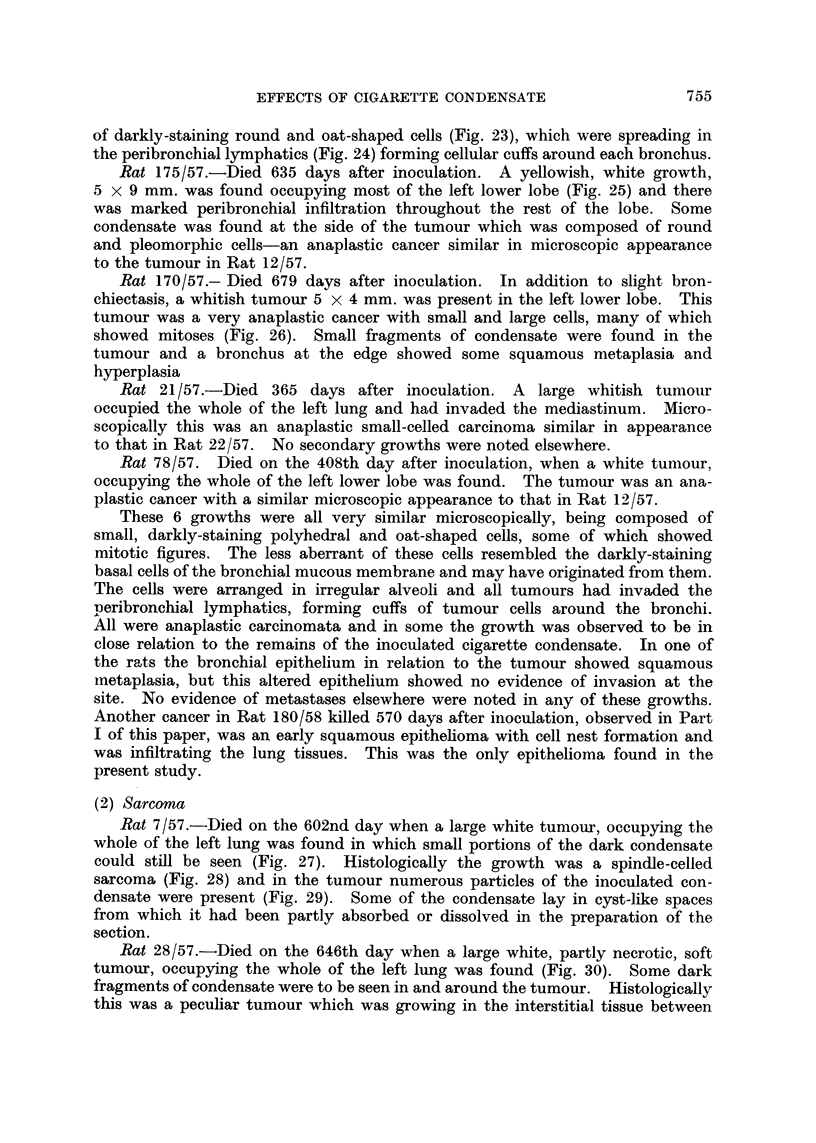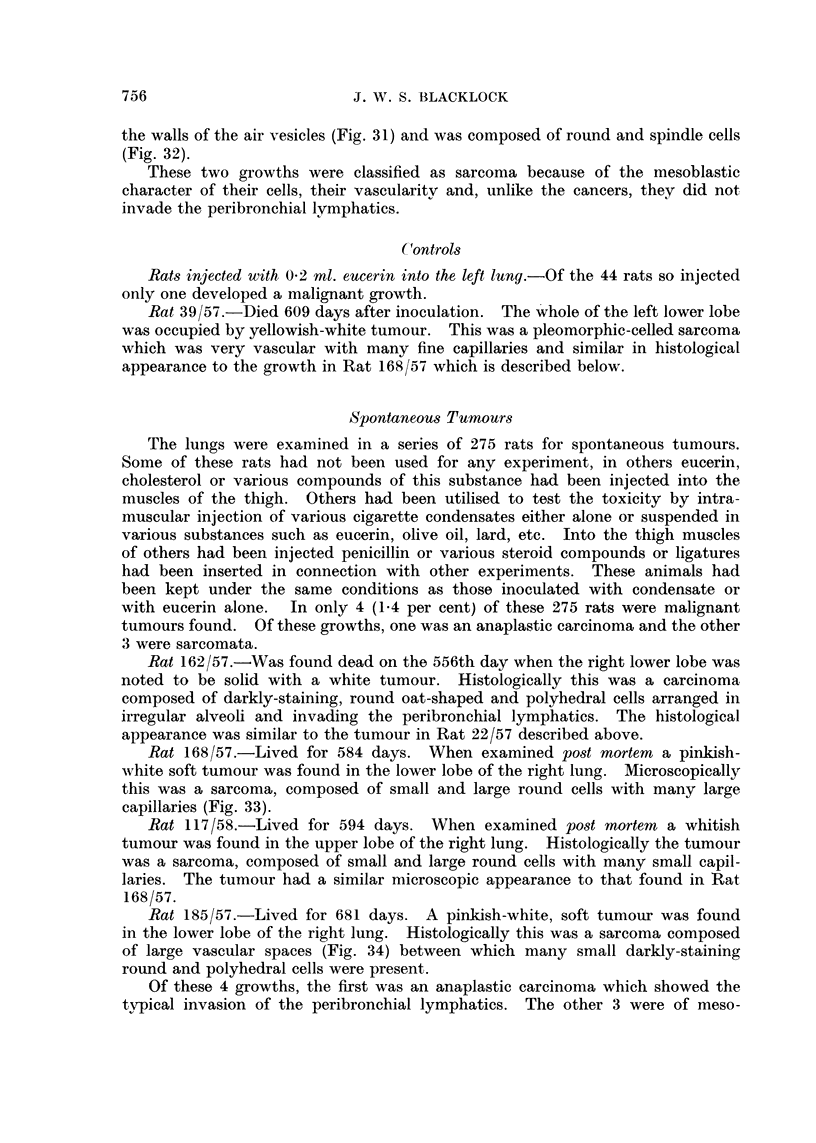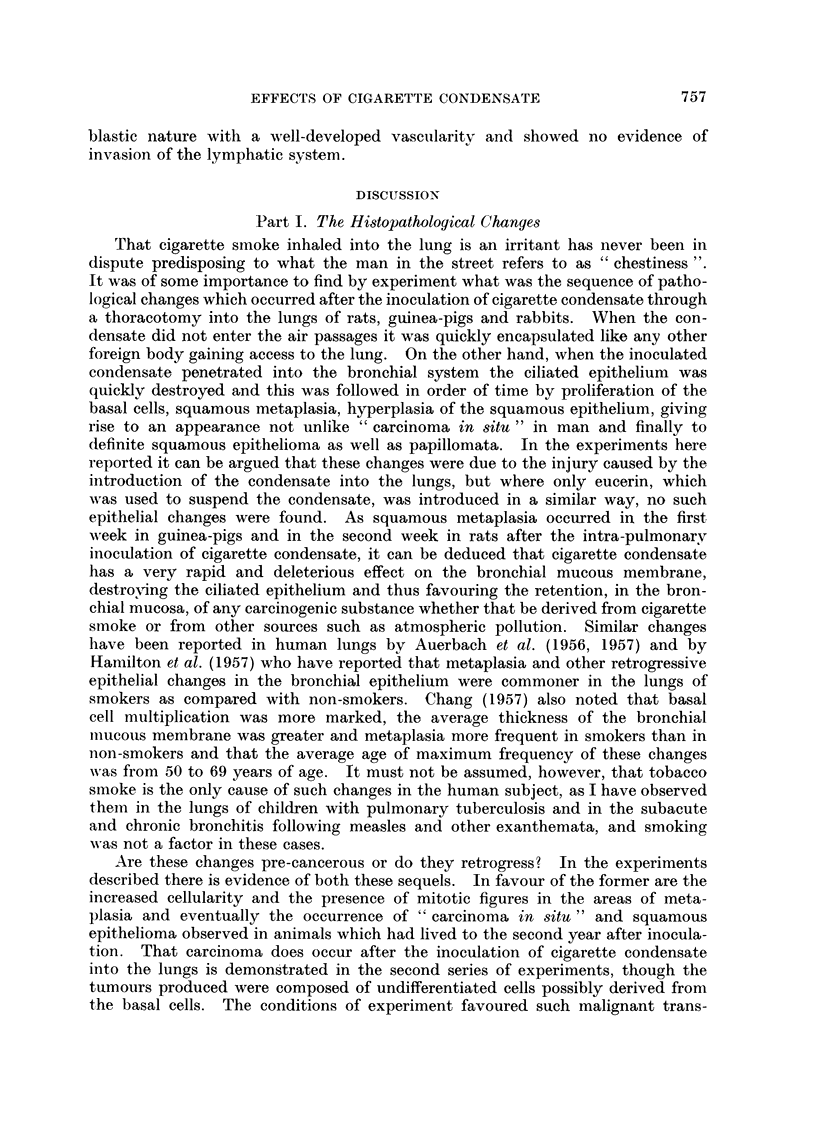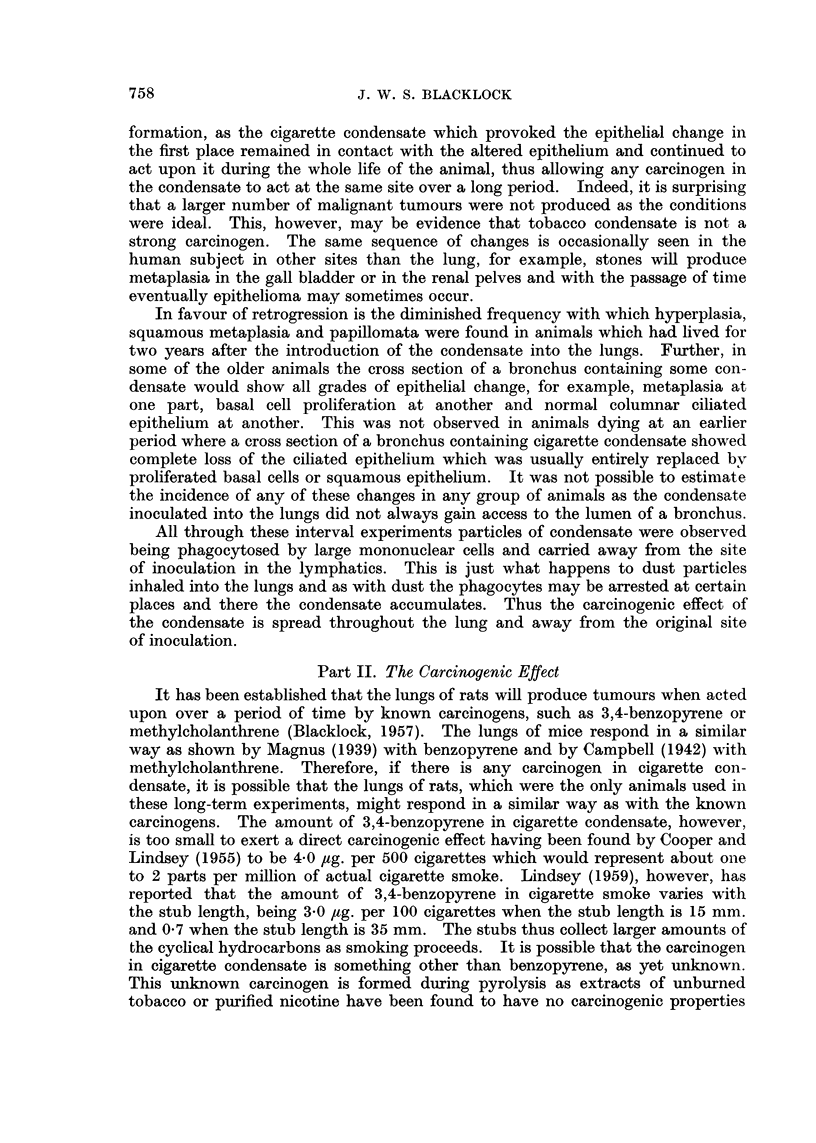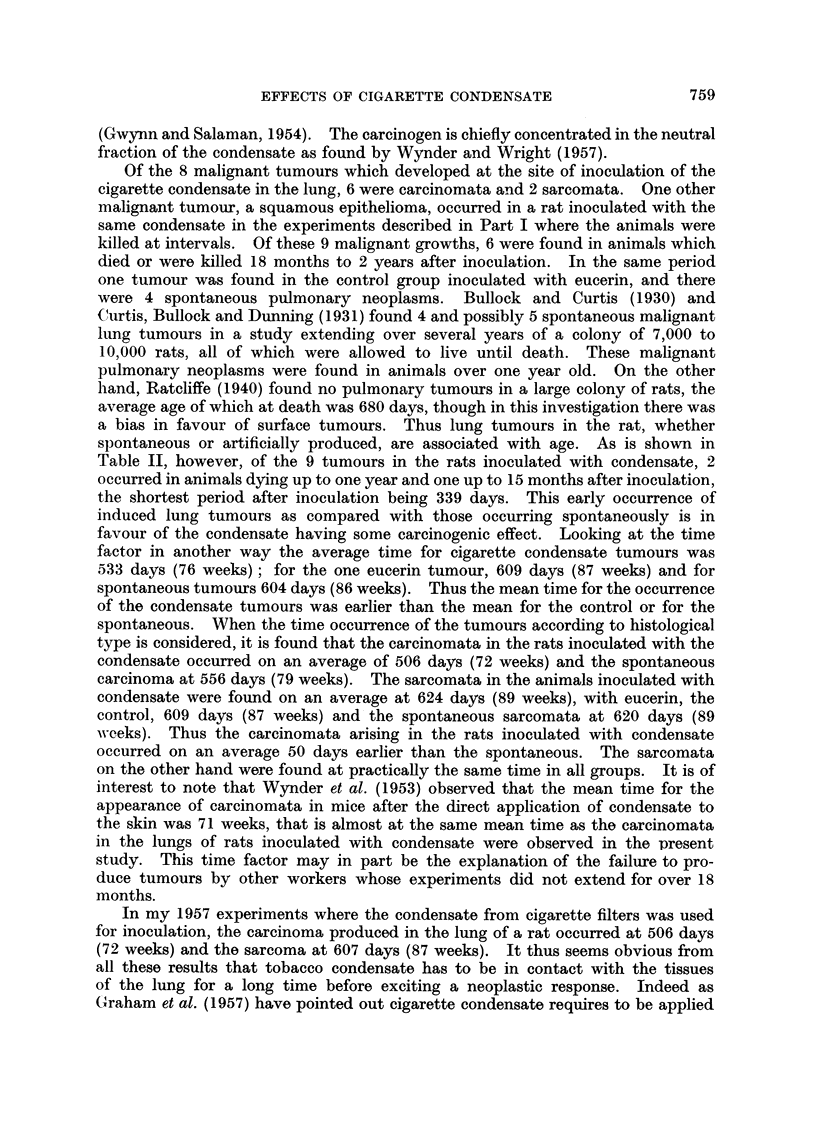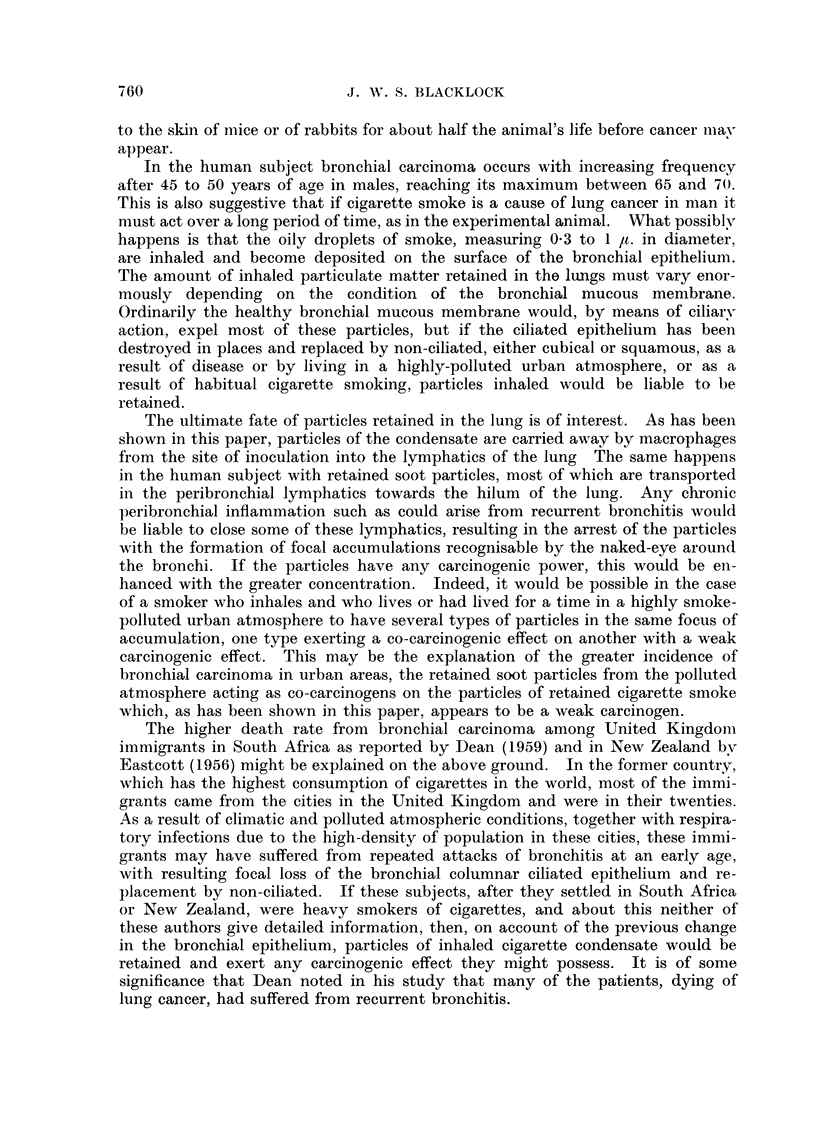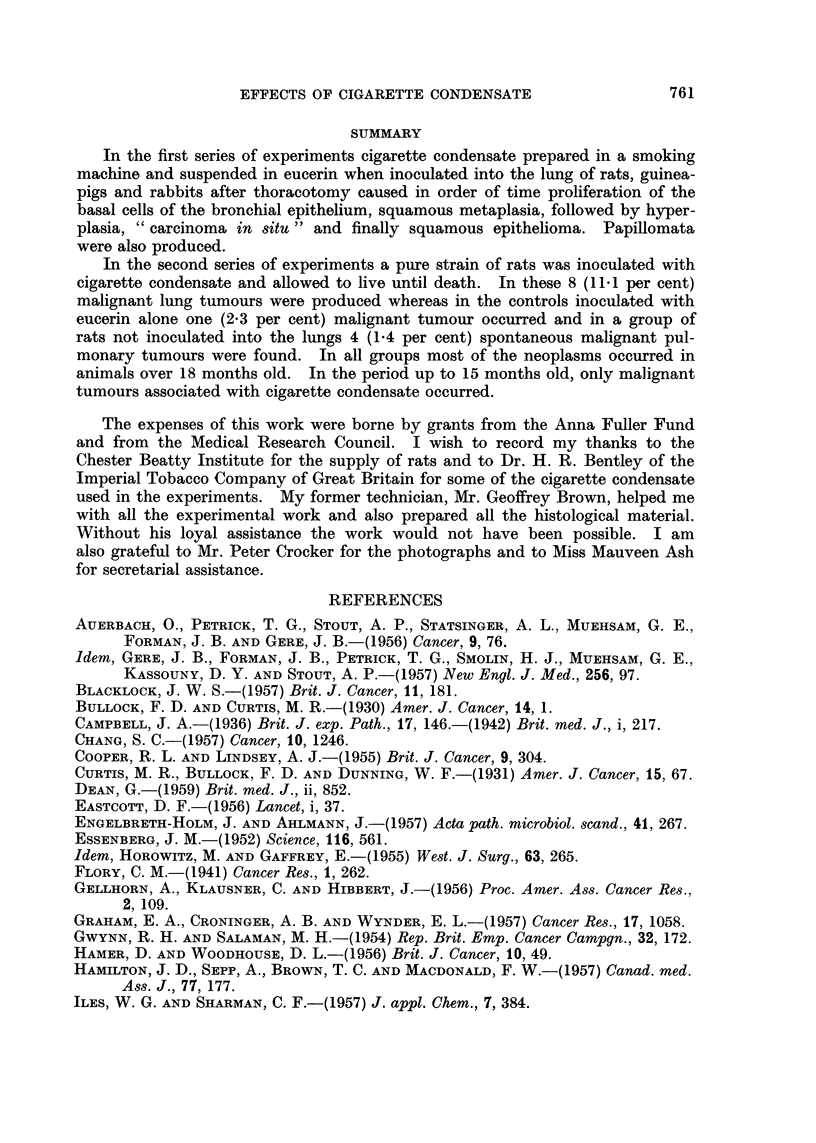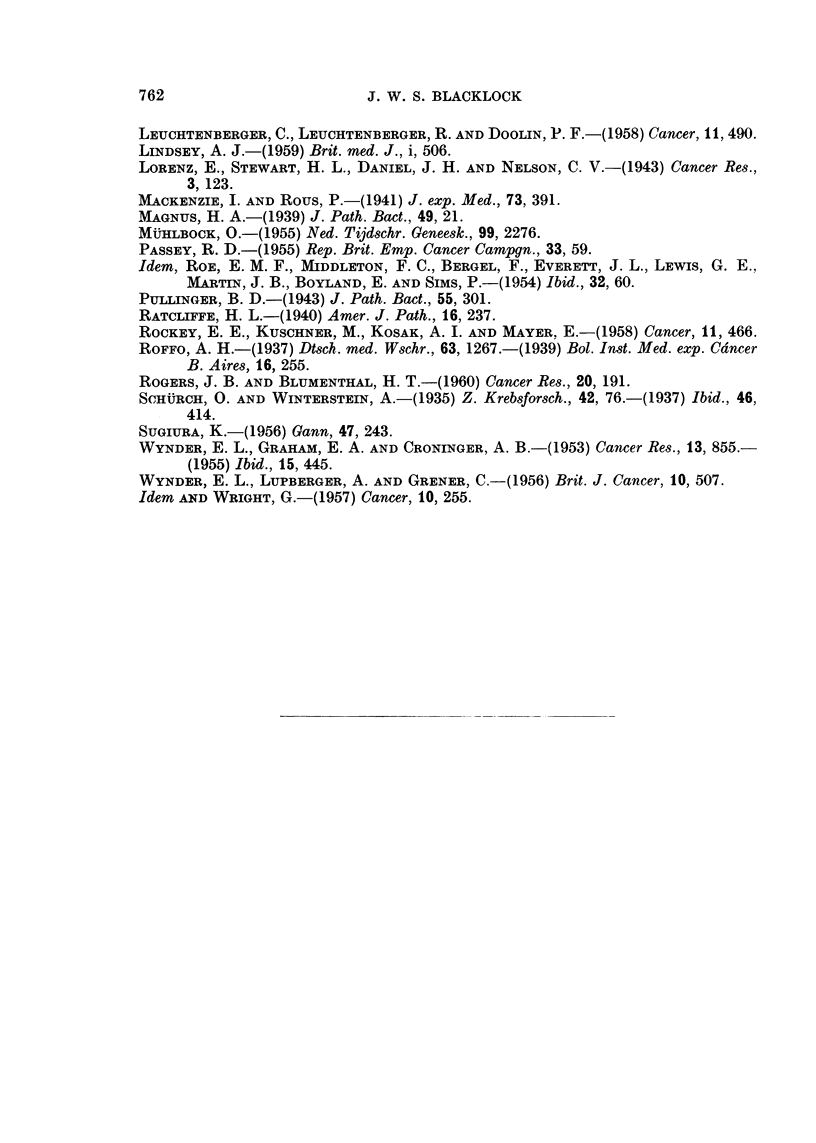# An Experimental Study of the Pathological Effects of Cigarette Condensate in the Lungs with Special Reference to Carcinogenesis

**DOI:** 10.1038/bjc.1961.85

**Published:** 1961-12

**Authors:** J. W. S. Blacklock

## Abstract

**Images:**


### AN EXPERIMENTAL STUDY OF THE PATHOLOGICAL EFFECTS OF CIGARETTE CONDENSATE IN THE LUNGS WITH SPECIAL REFERENCE TO CARCINOGENESIS

J. W. S. BLACKLOCK

*From the Department of Pathology, St. Bartholomew's Hospital, London, E.C.*1

Received for publication September 4, 1961

It has been demonstrated (Blacklock, 1957) that malignant tumours can be produced in the lungs of rats with various carcinogens, such as benzopyrene or methylcholanthrene, and in a small number of rats with the condensate from cigarette smoke. In view of the results with the condensate, it was desirable to repeat the experiments in a larger series of animals and in addition to study the histo-pathological changes in the lungs preceding the development of malignant tumours. To ensure that there was no other carcinogen at work in the course of the experiments here described, no experimental work with known carcinogens was carried out at the time in the laboratory concerned. Further, it is most unlikely that the benzopyrene content of the air at St. Bartholomew's Hospital, during the years this study was in progress, had any carcinogenic effect on the lungs of the experimental animals used, as my colleague, Dr. P. J Lawther, informs me that the mean benzopyrene content during that time was 2·5 $\mu$g./100m$^3$ and the mean smoke concentration was 0·15 mg./m$^3$.

Others have reported with varying degrees of success the experimental production of tumours by painting cigarette condensate on the skin of mice (Wynder, Graham and Croninger, 1953, 1955; Wynder, Lupberger and Grener, 1956; Sugiura, 1956; Engelbreth-Holm and Ahlmann, 1957). Similar experiments on mice extending over 18 to 19 months by Passey *et al.* (1954), Gwynn and Salaman (1954), Hamer and Woodhouse (1956) and Gellhorn, Klausner and Hibbert (1956) have yielded negative results. In rabbits Roffo (1937, 1939), Schürch and Winterstein (1935, 1937), Flory (1941) and Graham, Croninger and Wynder (1957) have produced tumours in the skin of the ear by the repeated application of tobacco tar, though of all these authors only Graham *et al.* used cigarette condensate, the others using distillates of various types of tobacco or of cigars. The amount of condensate used in such painting is gross and bears little relation to the amount that would be inhaled into the lung during smoking. The repeated painting over months of cigarette condensate on the skin may cause recurrent damage and even ulceration, as in Wynder's experiments, with subsequent repeated attempts at repair. This condition, at least in the human subject, predisposes to malignant change. In the experimental animal, as pointed out by Mackenzie and Rous (1941) and by Pullinger (1943), the additional factor of wound healing has a considerable effect on the yield of tumours by carcinogens. The above experiments on the carcinogenic effect of cigarette condensate on the skin of certain animals have been accepted by some and rejected by others. It remained to be proved, however, if the condensate had the same effect on the lungs. Cigarette smoke is an aerosol, the oily particles of which when first formed vary in size from 0·3 to 1 $\mu$. and particles of this size can, on inhalation, pass deeply into the lung. Accordingly rats and mice have been kept in an atmosphere of cigarette smoke for periods, though unlike in the human subject where the smoke is inhaled directly into the lungs through the mouth, the smoke in the animal passes through the nose and much of the smoke particles must be filtered out in the complicated nasal passages and never reaches the lungs. The results of keeping animals in an atmosphere of cigarette smoke have been varied, for example, Essenberg (1952) and Essenberg, Horowitz and Gaffrey (1955) found a greater number of papillary adenocarcinomata in pure strains of mice exposed to cigarette smoke than in the control group, and Mühlbock (1955), working with hybrid mice, found a higher incidence of lung tumours in those exposed to cigarette smoke than in the controls kept under the same conditions but not exposed to the smoke. Passey (1955 and personal communication) was unable to produce tumours in the lungs of hamsters or of rats exposed to cigarette smoke for periods of 12 to 20 months, and Lorenz *et al.* (1943) found the average number of neoplasms the same in the control mice as in those of the same strain exposed to tobacco smoke for 250 days. Campbell (1936) on the other hand found a slightly higher incidence of tumours in the lungs of mice exposed to tobacco smoke than in the controls, the tumours being more frequent about the end of the second year. Thus the results of experimental work in animals with cigarette condensate have so far been conflicting.

*The preparation of the cigarette condensate*

The condensate was prepared by smoking cigarettes of a popular brand and reputed to be composed of 60 per cent Virginian and 40 per cent Rhodesian tobacco in a smoking machine described by Cooper and Lindsey (1955). The condensate from 500 cigarettes was collected in acetone, which was evaporated at 50° C. and the viscous condensate was stored in a tightly stoppered glass bottle in the dark at 4° C. The chemical composition of condensate prepared in this manner and from the same brand of cigarettes has been reported by Cooper and Lindsey (1955) and contained 4 $\mu$g. of 3,4-benzopyrene per 500 cigarettes. In the experiments described here the nicotine was not removed as the chemical treatment involved might cause changes in the condensate.

*Operative procedure*

For most of the experiments male white rats 8 to 12 weeks old were used. These rats were all of the same strain and were bred at the Chester Beatty Institute in London. The same type of rat was used in the work reported in 1957. Guinea-pigs and rabbits were used for some experiments. The former were 3 to 4 months old and the latter 5 to 6 months when inoculated. Neither were of pure strains and were purchased from dealers.

In order to imitate human smoking the pure cigarette condensate prepared as above but warmed to reduce its viscosity was, at the outset of these experiments, injected down the trachea by means of a long needle with a blunt tip which reached to about the level of the bifurcation of the trachea of the rats. Most of the animals, however, died immediately of suffocation and intoxication or later from a septic pneumonia. As the mortality was so great this experiment was abandoned.

It was then decided, as in the 1957 experiments, to inject the condensate directly into the lungs after a thoracotomy had been performed. The needle having pierced the lung was directed towards the hilum in the hope that some of the condensate might gain access to a bronchus. When the condensate made as described above was warmed to allow it to pass easily through a fine needle and inoculated into the lung, much of it ran back through the needle track into the pleural sac and there were also symptoms of intoxication. It was obvious that some vehicle was required which would mix with the condensate, not alter it chemically and be of a consistency that would go through a fine needle and retain the condensate at the site of inoculation. It was found by experiment that eucerin* fulfilled these conditions and further this substance produced little reaction in the tissues. The eucerin also partly neutralised the toxic effect of the nicotine, probably because the alkaloid was liberated slowly from the mixture. When required for inoculation the appropriate amount of condensate was mixed with eucerin so that each 0·2 ml. of the mixture contained the condensate from approximately 40 cigarettes. Unless otherwise stated this amount was inoculated into the left lung of the rats, guinea-pigs and rabbits used for these experiments. In the rats submitted to this operation there was a 10 per cent mortality within 24 hours.

The condensate was used for inoculation within a few days of preparation and any remaining 15 days after preparation was discarded. But having used eucerin as a vehicle, it was desirable to test, as is reported later, what reactions it produced when introduced into the tissues and also to find if it had any carcinogenic effect.

It was realised that 0·2 ml. of the condensate-eucerin mixture was a large amount to inoculate into the lung, but it appeared from the results of the 1957 experiments that cigarette condensate was a weak carcinogen and, therefore, it was desirable to use a large dose and to allow it to act for as long as possible, that is, until the animal died or required to be killed on account of illness. Unlike in the painting of the skin with cigarette condensate, there was no repeated trauma which might be a factor in the production of a malignant tumour. In these lung experiments damage to the tissues occurred only once and was, as is afterwards shown, quickly followed by repair. If a tumour developed at the site of inoculation in the lung this could be considered as good evidence that the cigarette condensate was the cause.

### *Plan of experiments*

It was desirable to study firstly the histopathological reaction of the the lung to cigarette condensate in animals killed at various intervals and secondly any carcinogenic effect of the condensate in animals allowed to live their entire life span. These two studies, though closely related, are now considered separately.

### I. *The Histopathological Changes in the Lungs Caused by Cigarette Condensate*

These changes were studied in (a) rats, (b) guinea-pigs and (c) rabbits into the left lung of which 0·2 ml. cigarette condensate-eucerin mixture representing about 40 cigarettes was inoculated. As controls 0·2 ml. eucerin alone was in-

* The composition of the (hydrous) eucerin used in these experiments is described under the heading of Oily Cream or Unguentum Aquosum in *British Pharmacopoeia* 1958, p. 715.

oculated into the lungs of a series of rats. As a large amount of cigarette condensate was required for these experiments, Dr. H. R. Bentley of the Imperial Tobacco Company kindly supplied me with some condensate from the same brand of cigarettes as had been used in the preparation of the condensate in this laboratory. Dr. Bentley's condensate had been prepared in the automatic smoking machine described by Iles and Sharman (1957). About half the animals in this part of the work were inoculated with this condensate, the other half with that prepared in our own smoking machine as described above.

(a) *Rats*

The rats were killed at intervals of one week to over two years after inoculation. The number of animals killed at each interval varied slightly. The results produced depended on whether the condensate did or did not penetrate into the bronchial system. Owing to the experimental difficulties attending the inoculation when the thorax was open the condensate did not always enter the bronchi but sometimes remained in the lung around the air passages. When this occurred the inoculum was quickly encapsulated by granulation and later by fibrous tissue. With the phagocytosis and the solution of the eucerin used as a suspending agent, some small cyst-like spaces with fine fibrous walls resulted and in these brownish-red portions of condensate were seen. In such areas there was a slight accumulation of mononuclear leucocytes, some foam cells and a few giant cells (Fig. 1). Later some calcification took place and these areas were always sharply demarcated by a fibrous capsule from the surrounding tissues. Where, however, the condensate did gain access to the bronchi, the sequence of changes now to be described were noted.

1*st week*.—There were 13 rats in this group. Around the inoculum in the first few days there was a moderately acute reaction composed of polymorphs and some foam cells ; later there were more mononuclear leucocytes. Some of the capillaries which the inoculum had surrounded showed thrombosis. Where the inoculum had penetrated into a bronchus there was a reaction composed of polymorphonuclear and mononuclear leucocytes. The lining epithelium showed necrosis or had lost its columnar ciliated character and was becoming more cubical in shape or even flat and was several cells thick.

2*nd week*.—There were 14 animals in this group. It was during this week that squamous metaplasia and less often papillomatous change was noted in the bronchi into which the inoculum had penetrated. These changes were noted in 6 out of 14 animals killed at this time. The squamous change which involved the epithelium lining the whole circumference of the bronchus was sometimes fairly regular (Fig. 2) though several layers thick ; at other times it was more hyperplastic and epithelial processes extended into the underlying tissue, suggesting an early invasive tendency (Fig. 3) or where the hyperplasia was very marked giving rise to a condition not unlike what has been termed " carcinoma *in situ* " in man (Fig. 4). Papillomata were observed in the bronchi to which the condensate had gained access and were usually covered by a layer of cubical epithelium. The appearance of the metaplastic squamous change at such an early period after inoculation was remarkable. It was not until the seventeenth day after exposure to smoke of 6 to 8 cigarettes daily that Leuchtenberger, Leuchtenberger and Doolin (1958) observed atypical basal-celled hyperplasia in the bronchial epithelium of $CF_1$ albino mice. At a later date they observed epithelial dysplasia similar to " carcinoma *in situ* " in man as well as squamous metaplasia in a small number of mice. Rockey *et al.* (1958) by applying cigarette condensate to the medial surface of the left main bronchus, through an artificial tracheo-cutaneous fistula, noted transitional metaplasia in 2 dogs which had received 2 treatments each and which had survived for 2 and 3 days respectively, and squamous metaplasia in other 2 dogs which survived for 17 and 25 days and received 6 and 17 treatments respectively.

*3rd week.*—There were 7 animals in this group and the changes were similar to those in the second week though the proportion of cases showing squamous metaplasia was less and those found were more regular and less hyperplastic than formerly. The leucocytes around the inoculum were much less numerous than formerly and composed mostly of large mononuclear leucocytes.

*4th–16th weeks.*—There were 36 rats in this group and during this period the rats killed at weekly intervals showed a progressive increase of fibrous tissue around the inoculum. Phagocytosis of small particles of condensate by large mononuclear leucocytes was noted now with increasing frequency. These tar-laden phagocytes were passing out from the area of the inoculum into the surrounding lung tissue. Squamous metaplasia of the bronchial epithelium, or of the lining of large cysts, derived from bronchi which had been blocked with condensate or papillomata and later with desquamated epithelium, were much less frequent and papillomatous growths into the bronchi were not so common (Fig. 5). In a cross section of a small bronchus these papilloma almost completely occluded the lumen and the central stroma of the papilloma sometimes contained small portions of condensate around which there was a mononuclear cell reaction and some small giant cells (Fig. 6). Large cystic spaces, due to the blockage of bronchi, were occasionally noted and into these papillomata, partly covered with squamous epithelium, had grown (Fig. 7). In some cases there was evidence that the condensate, where small in amount, was being absorbed and where this had been in contact with the lining of a bronchus, the epithelium of which was undergoing metaplastic change, there was now some evidence that the cells were becoming more cubical in shape. In such a bronchus one could find all three types of epithelium, normal columnar ciliated, cubical and squamous. This reversal of the metaplastic change was noted from the 12th week after inoculation onwards. This was in marked contrast with what had been observed in the earlier weeks where bronchi containing condensate were lined by atypical epithelium of one type, basal, transitional or squamous.

*17th–52nd weeks.*—There were 72 rats in this group, 2 being killed at weekly intervals. During this period the fibrosis and mononuclear and giant cell reactions around the condensate remained unchanged, though in some cases there was a deposit of calcium salts in the inoculum and phagocytosis of small particles of the condensate was still frequently observed (Fig. 8). Some of the larger cysts of bronchial origin containing condensate had a squamous lining in which there were localised areas of hyperplasia which was invading the tissues outside (Fig. 9). The proportion of bronchi showing squamous metaplasia was less than in the earlier weeks.

*53rd–104th weeks.*—There were 34 animals in this group, 2 being killed every 3 weeks. Two years after inoculation, the site of injection of 0·2 ml. of tobacco condensate was still easily recognisable naked-eye, the lesion being very localised in the lung and sometimes showing an early deposit of calcium in the condensate

### EXPLANATION OF PLATES

FIG. 1.—Rat killed 14 days after inoculation. Encapsulation of brownish-red cigarette condensate by granulation tissue, mononuclear leucocytes and foreign body giant cells. ×95.

FIG. 2.—Rat killed 12 days after inoculation. Squamous metaplasia in epithelium lining the whole circumference of small bronchus. ×30 and ×110.

FIG. 3.—Rat killed 13 days after inoculation. Remains of fragments of condensate in spaces above a small bronchus showing irregular hyperplastic squamous metaplasia extending into surrounding tissue. ×55.

FIG. 4.—Rat killed 14 days after inoculation. Fragments of condensate and leucocytes in lumen of bronchus showing marked hyperplastic squamous metaplasia resembling " carcinoma *in situ* ". ×55.

FIG. 5.—Rat killed 56 days after inoculation. Papilloma growing into small bronchus. In the stroma of the papilloma are spaces which contained condensate. Multiplication of the basal cells is present both in the epithelium covering the papilloma and to a lesser extent in the bronchus. ×65.

FIG. 6.—Rat killed 56 days after inoculation. Cross section of a small bronchus, partly occluded by a papilloma, cut transversely. In the stroma of papilloma are spaces from which the condensate has been absorbed or dissolved and around these are some small giant cells. ×105.

FIG. 7.—Rat killed 112 days after inoculation. Part of cross section of a distended bronchus in which fragments of condensate are still present. Extending into the lumen is a papilloma partly covered by columnar ciliated epithelium and partly by squamous. The wall of the bronchus to the right of the papilloma is lined by squamous epithelium. ×50.

FIG. 8.—Rat killed 166 days after inoculation. Mononuclear leucocytes near site of inoculation which have phagocytosed small darkly staining particles of condensate. ×275.

FIG. 9.—Rat killed 263 days after inoculation. Part of a bronchus distended with condensate and squame cells. Squamous metaplasia of lining epithelium with extension into the peribronchial tissue. ×80.

FIG. 10.—Rat killed 491 days after inoculation. Part of a bronchus distended with condensate and squame cells. Squamous metaplasia of lining epithelium, extension into peribronchial tissue and formation of epithelial perles. ×95.

FIG. 11.—Rat killed 570 days after inoculation. Part of wall of distended bronchus showing remains of dark condensate (at top). Squamous epithelioma infiltrating lung tissue. ×50.

FIG. 12.—Guinea-pig killed 7 days after inoculation. Small bronchus with some condensate and leucocytes in lumen. The lining epithelium shows early squamous change. ×165.

FIG. 13.—Guinea-pig killed 13 days after inoculation. Portion of a bronchus distended with condensate (at the top) and some leucocytes. The lining epithelium has all undergone squamous metaplasia. ×72.

FIG. 14.—Guinea-pig killed 21 days after inoculation. Portion of the lumen of a distended bronchus containing condensate and leucocytes. Numerous papillomata covered with a cubical epithelium are growing from the wall. ×60.

FIG. 15.—Guinea-pig killed 15 days after inoculation. Adenomatous formation at the edge of site of inoculum. The lining cells show much irregularity and mitotic figures are present in some. ×160.

FIG. 16.—Guinea-pig killed 275 days after inoculation. Papillomata growing into a bronchus distended with condensate to show the cubical character of covering epithelium. ×130.

FIG. 17.—Guinea-pig killed 285 days after inoculation. A sessile papilloma growing into a distended bronchus. The growth is covered with a very hyperplastic squamous epithelium and in the stroma are numerous adenomata. ×35.

FIG. 18.—Guinea-pig killed 295 days after inoculation. Portion of one of the main bronchi with cartilage in wall showing very marked hyperplasia of the basal cells. ×50.

FIG. 19.—Guinea-pig killed 275 days after inoculation. Portion of wall of a bronchus showing squamous change in the lining epithelium and early sessile papilloma formation. ×50.

FIG. 20.—Rabbit killed 50 days after inoculation. Portion of wall of a bronchus with condensate in the lumen above. The lining epithelium shows marked cellular proliferation resembling " carcinoma *in situ* ". ×55.

FIG. 21.—Rat died 339 days after inoculation. A pleomorphic celled cancer with an alveolar arrangement. ×210.

FIG. 22. Rat died 550 days after inoculation. A vertical section through left lung showing white tumour which is extending along peribronchial lymphatics. Dark portions of cigarette condensate are present at the centre of the growth and at the upper and lower edges. The left ventricle cut transversely is to the left and above. ×1·2.

(*continued on p.* 751)

1

10

Blacklock.

2

3

4

5

6

7

8

9

11

12

13

Blacklock.

14

15

16

17

18

19

20

21

22

23

24

25

26

27

28

29

30

31

32

33

34

or in the encapsulating fibrous tissue. Squamous metaplasia was still noted in the bronchi to which condensate had gained access, though less often than formerly. Owing to the blockage of bronchi by condensate or papillomata or both any metaplastic change of the normal epithelium to a squamous type had resulted in much desquamation of squame cells into the lumen which caused distension. As a result large cysts were formed containing the remains of the condensate mixed with squame cells. In some of these cysts the metaplastic epithelium was hyperplastic and extended down into the underlying tissue with the formation of epithelial perles (Fig. 10) or even invasion of the deeper tissues. In 2 animals showing such change one was killed at 491 days (70 weeks) and the other (180/58) at 570 days (81 weeks) and it was debatable on first examination if the condition was definitely malignant. With the study of further sections the first of these has been regarded as non-malignant, though it might have become so if the animal had been allowed to live. The second, however, in addition to more formation of epithelial perles showed definite invasion in all the sections examined and has been classified as an early squamous epithelioma (Fig. 11).

At this period there was more evidence that in some bronchi the metaplastic change in the epithelium was being reversed as it was possible to find in the circumference of a dilated bronchus squamous epithelium, with transition to cubical and finally normal columnar ciliated. It was only towards the end of the second year that adenomatous lesions were noted in the vicinity of the inoculum in the lungs of rats.

(b) *Guinea-pigs*

As in the experiments with rats 0·2 ml. of the cigarette condensate-eucerin mixture was inoculated into the left lung after a thoracotomy had been performed.

---

(*continued from p.* 750.)

FIG. 23.—Microscopic appearance of gross tumour shown in Fig. 22. The tumour is composed of darkly staining small round and oat-shaped cells. ×210.

FIG. 24.—Microscopic appearance of tumour shown in Fig. 22 to show peribronchial spread. ×6·5.

FIG. 25.—Rat died 635 days after inoculation. Vertical section through tumour in left lower lobe. ×5.

FIG. 26.—Rat died 679 days after inoculation. A pleomorphic celled carcinoma composed of small and large round cells and some polygonal cells. Some cells show mitosis. ×210.

FIG. 27.—Rat died 602 days after inoculation. Vertical section through left lung almost completely replaced by whitish tumour, embedded in which are some dark fragments of condensate. Heart is to left. ×1·2.

FIG. 28.—Microscopic appearance of tumour in Fig. 27—a spindle-celled sarcoma. ×210.

FIG. 29.—Microscopic appearance of tumour in Fig. 27 showing fragments of dark condensate in tumour and in a cystic space to right. ×55.

FIG. 30.—Rat died 646 days after inoculation. Vertical section through left lung which has been replaced by a white tumour which is breaking down. Some dark fragments of condensate are visible in the tumour. The left ventricle cut transversely is above and to left. ×1·2.

FIG. 31.—Microscopic appearance of gross tumour shown in Fig. 30. A sarcoma which is growing in the inter-alveolar tissue compressing the air vesicles. ×52.

FIG. 32.—A higher power view of Fig. 31 to show the imperfect spindle-shaped character of the cells. ×190.

FIG. 33.—Rat died, aged 584 days. A round-celled sarcoma with some fine intercellular reticulin and capillary vessels. ×190.

FIG. 34.—Rat died, aged 681 days. An angio-sarcoma composed of many large vascular sinusoids between which are darkly staining round cells. ×52.

The animals thereafter were killed at intervals and the lesions studied. There was no attempt to produce tumours in guinea-pigs as these animals are comparatively refractory to carcinogenic agents and no spontaneous tumours were found in a review of the literature by Rogers and Blumenthal (1960).

1*st week*.—There were 16 animals in this group. The condensate had produced some localised necrosis at the site of inoculation and around this were polymorphonuclear leucocytes and macrophages. From the 5th to the 7th day, dedifferentiation of the lung epithelium was noted and on the 7th day after inoculation actual squamous change (Fig. 12), whereas in rats this was not noted until the second week.

2*nd week*.—There were 19 animals in this group. The cell reaction was now more mononuclear and granulation tissue showing well-developed fibrous tissue was present around the condensate. Where in contact with the condensate there was proliferation of the basal cells in the bronchial epithelium with some hyperplasia and in some cases complete squamous change was also present (Fig. 13).

3*rd week*.—There were 9 animals in this group. The condensate was now well encapsulated by fibrous tissue : the cellular reaction was still mononuclear, with in addition fairly numerous foreign-body giant cells. Where the condensate had come in contact with the epithelium of a bronchus, the changes noted in the second week were still evident in addition to papillomatous change (Fig. 14) and adenomata which were lined by rather anaplastic cells some of which showed mitosis (Fig. 15). In the metaplastic squamous epithelium mitotic figures were frequently observed.

4*th–5th weeks*.—There were 11 animals in this group. The changes were very similar to those in the 3rd week, except for the increasing number of giant cells around the lesions. Dedifferentiation and papillomatous outgrowths of the bronchial epithelium and adenoma and papilloma and squamous metaplasia were all as frequent as at the earlier periods.

6*th–52nd weeks*—There were 33 animals in this group. At this late period the most noteworthy changes were the increasing amount of hyperplasia of the bronchial epithelium where condensate was present. All the changes noted above were still observed, though papillomata were more frequent and larger, some being covered with cubical epithelium (Fig. 16) and others with squamous (Fig. 17). Hyperplastic changes were still observed in the bronchial epithelium such as proliferation of the basal cells (Fig. 18) and as in the rats metaplasia to a hyperplastic squamous type (Fig. 19). Giant cells phagocytosing the condensate were also frequently seen.

(c) *Rabbits*

Similar experiments to those in rats and in guinea-pigs were performed in a small series of rabbits, 0·2 ml. of cigarette condensate-eucerin mixture being introduced into the left lung after thoracotomy. All the changes were similar to those reported in the guinea-pigs, though some were noteworthy. For example, in one rabbit killed 50 days after inoculation, the epithelium of the bronchus to which the condensate had gained access showed all degrees of epithelial change. Part was normal where in contact with the condensate and part showed extreme hyperplasia of the basal cells and dedifferentiation, forming what has been described as " carcinoma *in situ* " (Fig. 20) with intermediate changes of simple dedifferentiation to a cuboidal epithelium and squamous metaplasia.

*Controls*

0·2 ml. of eucerin was inoculated into the left lung of a series of rats to observe the effects of this substance which had been used as a vehicle in which to suspend the tobacco condensate. The animals were killed at intervals after inoculation.

In the first week there was an accumulation of foam cells around the eucerin with slight growth of fine granulation tissue. In the second week the eucerin was contained in many small spaces separated by fine fibrous tissue. This lesion remained unaltered throughout the rest of the time the animals lived up to 691 days. In the second year slight calcification was observed in the contents of these spaces. Apart from these changes eucerin caused no other pathological changes in the lungs of rats and even where it had gained access to bronchi, the epithelium remained normal and only some foam cells were found in the lumen.

### II. *The Carcinogenic Effect of Cigarette Condensate in the Lung*

The Chester Beatty strain of rats were used in this part of the work and after inoculation they were allowed to live until death or until they appeared acutely ill and required to be killed. Into the left lung of these rats, 8 to 12 weeks old, 0·2 ml. cigarette-eucerin mixture, prepared as previously described, was inoculated. For controls a series of rats of the same strain were inoculated into the left lung with 0·2 ml. eucerin alone. A number of rats which were not inoculated in the lung were examined for the presence of spontaneous pulmonary tumours.

In 72 rats which had been inoculated with cigarette condensate the experiments were suitable for analysis. Of these 8 (11·1 per cent) had developed malignant tumours—6 carcinomata and 2 sarcomata—at the site of inoculation. In the first section of this work in which the histo-pathological effects of cigarette condensate are considered, there was one rat (180/58) which when killed on the 570th day (81 weeks) after inoculation showed an early squamous epithelioma at the site of injection. This result has been excluded for the present as its inclusion would upset the incidence of tumours in this series where the animals were allowed to live their entire life span. It has, however, been included in Table II. Of the 44 control rats inoculated with eucerin alone, 1 (2·3 per cent) developed a malignant tumour—a sarcoma—at the site of inoculation. The number of spontaneous tumours found in the lungs of a series of 275 rats amounted to 4 (1·4 per cent)—one carcinoma and 3 sarcomata (Table I).

The highest incidence of malignant tumours was thus in the group inoculated in the lung with cigarette condensate. In the first year 2 out of 13 of these rats

TABLE I.—*Incidence of Malignant Tumours in Lung* (a) *After Inoculation of Cigarette Condensate or* (b) *of Eucerin and of Spontaneous Tumours*

| | —12 months | | | | —24 months | | | | Totals | |
|---|---|---|---|---|---|---|---|---|---|---|
| Experiment | Number of rats | Days: Lived | Days: Average | Malignant tumours | Number of rats | Days: Lived | Days: Average | Malignant tumours | Rats | Malignant tumours |
| Cigarette condensate into left lung | 13 | 182–364 | 277 | 2 (15·4%) | 59 | 421–729 | 557 | 6 (10·2%) | 72 | 8 (11·1%) |
| Eucerin into left lung | 7 | 114–179 | 141 | 0 | 37 | 416–690 | 553 | 1 (2·7%) | 44 | 1 (2·3%) |
| Spontaneous lung tumours | 80 | 141–364 | 245 | 0 | 195 | 399–726 | 555 | 4 (2·05%) | 275 | 4 (1·4%) |

(15·4 per cent) developed malignant tumours whereas no malignant tumours were found in the 7 rats in the control series which died in the first year and no spontaneous tumours occurred in the 80 rats which died during this time. In the second year, 6 of 59 (10·2 per cent) rats inoculated with cigarette condensate developed lung tumours, 1 out of 37 (2·7 per cent) inoculated with eucerin alone and 4 of 195 (2·05 per cent) developed spontaneous lung tumours. Most of the malignant growths were observed between 18 and 21 months in all groups (Table II).

TABLE II.—*Types of Malignant Tumours and Times of Occurrence*

| | Cigarette condensate | | Controls: Eucerin | | Controls: Spontaneous | |
|---|---|---|---|---|---|---|
| Months | Tumour | Days after inoculation | Tumour | Days after inoculation | Tumour | Days lived |
| −12 | Cancer<br>Cancer | 339<br>365 | 0 | .. | 0 | .. |
| −15 | Cancer | 408 | 0 | .. | 0 | .. |
| −18 | 0 | .. | 0 | .. | 0 | .. |
| −21 | Cancer<br>*Cancer<br>Sarcoma<br>Cancer | 550<br>570<br>602<br>635 | Sarcoma | 609 | Cancer<br>Sarcoma<br>Sarcoma | 556<br>584<br>594 |
| −24 | Sarcoma<br>Cancer | 646<br>679 | 0 | .. | Sarcoma | 681 |
| Total | 7 cancers<br>2 sarcoma | 339–679<br>602–646 | 1 sarcoma | 609 | 1 cancer<br>3 sarcoma | 556<br>584–681 |

* A squamous epithelioma which occurred in the experiments in Part I and not included in Part II

### *Types of Malignant Growths*

*Rats inoculated with cigarette condensate.*—The amount of condensate injected into each rat was approximately from 40 cigarettes. There were 8 malignant growths of which 6 were carcinomata and 2 sarcomata.

### (1) *Carcinoma*

*Rat* 12/57.—Died 339 days after inoculation. The lungs showed slight bronchiectasis and in the left lung a yellowish nodule (5 × 7 mm.) was noted. On section this was a pleomorphic-celled carcinoma (Fig. 21). This tumour was in close relation to the site of the injected condensate and eucerin, which had been dissolved during the preparation of the section, and in the vicinity many foam cells were present in addition to the malignant cells.

*Rat* 22/57.—Died 550 days after inoculation. A small, whitish growth, 6 × 8 mm., was noted in the left lower lobe with the remains of the condensate in the centre of the tumour around the edges of which there was peribronchial infiltration (Fig. 22). Microscopically the remains of the condensate, consisting of dark brown pigment with some spaces from which the eucerin had been dissolved, were found in immediate relation to the tumour, an anaplastic cancer composed of darkly-staining round and oat-shaped cells (Fig. 23), which were spreading in the peribronchial lymphatics (Fig. 24) forming cellular cuffs around each bronchus.

*Rat* 175/57.—Died 635 days after inoculation. A yellowish, white growth, 5 × 9 mm. was found occupying most of the left lower lobe (Fig. 25) and there was marked peribronchial infiltration throughout the rest of the lobe. Some condensate was found at the side of the tumour which was composed of round and pleomorphic cells—an anaplastic cancer similar in microscopic appearance to the tumour in Rat 12/57.

*Rat* 170/57.– Died 679 days after inoculation. In addition to slight bronchiectasis, a whitish tumour 5 × 4 mm. was present in the left lower lobe. This tumour was a very anaplastic cancer with small and large cells, many of which showed mitoses (Fig. 26). Small fragments of condensate were found in the tumour and a bronchus at the edge showed some squamous metaplasia and hyperplasia

*Rat* 21/57.—Died 365 days after inoculation. A large whitish tumour occupied the whole of the left lung and had invaded the mediastinum. Microscopically this was an anaplastic small-celled carcinoma similar in appearance to that in Rat 22/57. No secondary growths were noted elsewhere.

*Rat* 78/57. Died on the 408th day after inoculation, when a white tumour, occupying the whole of the left lower lobe was found. The tumour was an anaplastic cancer with a similar microscopic appearance to that in Rat 12/57.

These 6 growths were all very similar microscopically, being composed of small, darkly-staining polyhedral and oat-shaped cells, some of which showed mitotic figures. The less aberrant of these cells resembled the darkly-staining basal cells of the bronchial mucous membrane and may have originated from them. The cells were arranged in irregular alveoli and all tumours had invaded the peribronchial lymphatics, forming cuffs of tumour cells around the bronchi. All were anaplastic carcinomata and in some the growth was observed to be in close relation to the remains of the inoculated cigarette condensate. In one of the rats the bronchial epithelium in relation to the tumour showed squamous metaplasia, but this altered epithelium showed no evidence of invasion at the site. No evidence of metastases elsewhere were noted in any of these growths. Another cancer in Rat 180/58 killed 570 days after inoculation, observed in Part I of this paper, was an early squamous epithelioma with cell nest formation and was infiltrating the lung tissues. This was the only epithelioma found in the present study.

(2) *Sarcoma*

*Rat* 7/57.—Died on the 602nd day when a large white tumour, occupying the whole of the left lung was found in which small portions of the dark condensate could still be seen (Fig. 27). Histologically the growth was a spindle-celled sarcoma (Fig. 28) and in the tumour numerous particles of the inoculated condensate were present (Fig. 29). Some of the condensate lay in cyst-like spaces from which it had been partly absorbed or dissolved in the preparation of the section.

*Rat* 28/57.—Died on the 646th day when a large white, partly necrotic, soft tumour, occupying the whole of the left lung was found (Fig. 30). Some dark fragments of condensate were to be seen in and around the tumour. Histologically this was a peculiar tumour which was growing in the interstitial tissue between the walls of the air vesicles (Fig. 31) and was composed of round and spindle cells (Fig. 32).

These two growths were classified as sarcoma because of the mesoblastic character of their cells, their vascularity and, unlike the cancers, they did not invade the peribronchial lymphatics.

### *Controls*

*Rats injected with* 0·2 *ml. eucerin into the left lung.*—Of the 44 rats so injected only one developed a malignant growth.

*Rat* 39/57.—Died 609 days after inoculation. The whole of the left lower lobe was occupied by yellowish-white tumour. This was a pleomorphic-celled sarcoma which was very vascular with many fine capillaries and similar in histological appearance to the growth in Rat 168/57 which is described below.

### *Spontaneous Tumours*

The lungs were examined in a series of 275 rats for spontaneous tumours. Some of these rats had not been used for any experiment, in others eucerin, cholesterol or various compounds of this substance had been injected into the muscles of the thigh. Others had been utilised to test the toxicity by intramuscular injection of various cigarette condensates either alone or suspended in various substances such as eucerin, olive oil, lard, etc. Into the thigh muscles of others had been injected penicillin or various steroid compounds or ligatures had been inserted in connection with other experiments. These animals had been kept under the same conditions as those inoculated with condensate or with eucerin alone. In only 4 (1·4 per cent) of these 275 rats were malignant tumours found. Of these growths, one was an anaplastic carcinoma and the other 3 were sarcomata.

*Rat* 162/57.—Was found dead on the 556th day when the right lower lobe was noted to be solid with a white tumour. Histologically this was a carcinoma composed of darkly-staining, round oat-shaped and polyhedral cells arranged in irregular alveoli and invading the peribronchial lymphatics. The histological appearance was similar to the tumour in Rat 22/57 described above.

*Rat* 168/57.—Lived for 584 days. When examined *post mortem* a pinkish-white soft tumour was found in the lower lobe of the right lung. Microscopically this was a sarcoma, composed of small and large round cells with many large capillaries (Fig. 33).

*Rat* 117/58.—Lived for 594 days. When examined *post mortem* a whitish tumour was found in the upper lobe of the right lung. Histologically the tumour was a sarcoma, composed of small and large round cells with many small capillaries. The tumour had a similar microscopic appearance to that found in Rat 168/57.

*Rat* 185/57.—Lived for 681 days. A pinkish-white, soft tumour was found in the lower lobe of the right lung. Histologically this was a sarcoma composed of large vascular spaces (Fig. 34) between which many small darkly-staining round and polyhedral cells were present.

Of these 4 growths, the first was an anaplastic carcinoma which showed the typical invasion of the peribronchial lymphatics. The other 3 were of mesoblastic nature with a well-developed vascularity and showed no evidence of invasion of the lymphatic system.

### DISCUSSION

### Part I. *The Histopathological Changes*

That cigarette smoke inhaled into the lung is an irritant has never been in dispute predisposing to what the man in the street refers to as " chestiness ". It was of some importance to find by experiment what was the sequence of pathological changes which occurred after the inoculation of cigarette condensate through a thoracotomy into the lungs of rats, guinea-pigs and rabbits. When the condensate did not enter the air passages it was quickly encapsulated like any other foreign body gaining access to the lung. On the other hand, when the inoculated condensate penetrated into the bronchial system the ciliated epithelium was quickly destroyed and this was followed in order of time by proliferation of the basal cells, squamous metaplasia, hyperplasia of the squamous epithelium, giving rise to an appearance not unlike " carcinoma *in situ* " in man and finally to definite squamous epithelioma as well as papillomata. In the experiments here reported it can be argued that these changes were due to the injury caused by the introduction of the condensate into the lungs, but where only eucerin, which was used to suspend the condensate, was introduced in a similar way, no such epithelial changes were found. As squamous metaplasia occurred in the first week in guinea-pigs and in the second week in rats after the intra-pulmonary inoculation of cigarette condensate, it can be deduced that cigarette condensate has a very rapid and deleterious effect on the bronchial mucous membrane, destroying the ciliated epithelium and thus favouring the retention, in the bronchial mucosa, of any carcinogenic substance whether that be derived from cigarette smoke or from other sources such as atmospheric pollution. Similar changes have been reported in human lungs by Auerbach *et al.* (1956, 1957) and by Hamilton *et al.* (1957) who have reported that metaplasia and other retrogressive epithelial changes in the bronchial epithelium were commoner in the lungs of smokers as compared with non-smokers. Chang (1957) also noted that basal cell multiplication was more marked, the average thickness of the bronchial mucous membrane was greater and metaplasia more frequent in smokers than in non-smokers and that the average age of maximum frequency of these changes was from 50 to 69 years of age. It must not be assumed, however, that tobacco smoke is the only cause of such changes in the human subject, as I have observed them in the lungs of children with pulmonary tuberculosis and in the subacute and chronic bronchitis following measles and other exanthemata, and smoking was not a factor in these cases.

Are these changes pre-cancerous or do they retrogress? In the experiments described there is evidence of both these sequels. In favour of the former are the increased cellularity and the presence of mitotic figures in the areas of metaplasia and eventually the occurrence of " carcinoma *in situ* " and squamous epithelioma observed in animals which had lived to the second year after inoculation. That carcinoma does occur after the inoculation of cigarette condensate into the lungs is demonstrated in the second series of experiments, though the tumours produced were composed of undifferentiated cells possibly derived from the basal cells. The conditions of experiment favoured such malignant transformation, as the cigarette condensate which provoked the epithelial change in the first place remained in contact with the altered epithelium and continued to act upon it during the whole life of the animal, thus allowing any carcinogen in the condensate to act at the same site over a long period. Indeed, it is surprising that a larger number of malignant tumours were not produced as the conditions were ideal. This, however, may be evidence that tobacco condensate is not a strong carcinogen. The same sequence of changes is occasionally seen in the human subject in other sites than the lung, for example, stones will produce metaplasia in the gall bladder or in the renal pelves and with the passage of time eventually epithelioma may sometimes occur.

In favour of retrogression is the diminished frequency with which hyperplasia, squamous metaplasia and papillomata were found in animals which had lived for two years after the introduction of the condensate into the lungs. Further, in some of the older animals the cross section of a bronchus containing some condensate would show all grades of epithelial change, for example, metaplasia at one part, basal cell proliferation at another and normal columnar ciliated epithelium at another. This was not observed in animals dying at an earlier period where a cross section of a bronchus containing cigarette condensate showed complete loss of the ciliated epithelium which was usually entirely replaced by proliferated basal cells or squamous epithelium. It was not possible to estimate the incidence of any of these changes in any group of animals as the condensate inoculated into the lungs did not always gain access to the lumen of a bronchus.

All through these interval experiments particles of condensate were observed being phagocytosed by large mononuclear cells and carried away from the site of inoculation in the lymphatics. This is just what happens to dust particles inhaled into the lungs and as with dust the phagocytes may be arrested at certain places and there the condensate accumulates. Thus the carcinogenic effect of the condensate is spread throughout the lung and away from the original site of inoculation.

### Part II. *The Carcinogenic Effect*

It has been established that the lungs of rats will produce tumours when acted upon over a period of time by known carcinogens, such as 3,4-benzopyrene or methylcholanthrene (Blacklock, 1957). The lungs of mice respond in a similar way as shown by Magnus (1939) with benzopyrene and by Campbell (1942) with methylcholanthrene. Therefore, if there is any carcinogen in cigarette condensate, it is possible that the lungs of rats, which were the only animals used in these long-term experiments, might respond in a similar way as with the known carcinogens. The amount of 3,4-benzopyrene in cigarette condensate, however, is too small to exert a direct carcinogenic effect having been found by Cooper and Lindsey (1955) to be 4·0 $\mu$g. per 500 cigarettes which would represent about one to 2 parts per million of actual cigarette smoke. Lindsey (1959), however, has reported that the amount of 3,4-benzopyrene in cigarette smoke varies with the stub length, being 3·0 $\mu$g. per 100 cigarettes when the stub length is 15 mm. and 0·7 when the stub length is 35 mm. The stubs thus collect larger amounts of the cyclical hydrocarbons as smoking proceeds. It is possible that the carcinogen in cigarette condensate is something other than benzopyrene, as yet unknown. This unknown carcinogen is formed during pyrolysis as extracts of unburned tobacco or purified nicotine have been found to have no carcinogenic properties

(Gwynn and Salaman, 1954). The carcinogen is chiefly concentrated in the neutral fraction of the condensate as found by Wynder and Wright (1957).

Of the 8 malignant tumours which developed at the site of inoculation of the cigarette condensate in the lung, 6 were carcinomata and 2 sarcomata. One other malignant tumour, a squamous epithelioma, occurred in a rat inoculated with the same condensate in the experiments described in Part I where the animals were killed at intervals. Of these 9 malignant growths, 6 were found in animals which died or were killed 18 months to 2 years after inoculation. In the same period one tumour was found in the control group inoculated with eucerin, and there were 4 spontaneous pulmonary neoplasms. Bullock and Curtis (1930) and Curtis, Bullock and Dunning (1931) found 4 and possibly 5 spontaneous malignant lung tumours in a study extending over several years of a colony of 7,000 to 10,000 rats, all of which were allowed to live until death. These malignant pulmonary neoplasms were found in animals over one year old. On the other hand, Ratcliffe (1940) found no pulmonary tumours in a large colony of rats, the average age of which at death was 680 days, though in this investigation there was a bias in favour of surface tumours. Thus lung tumours in the rat, whether spontaneous or artificially produced, are associated with age. As is shown in Table II, however, of the 9 tumours in the rats inoculated with condensate, 2 occurred in animals dying up to one year and one up to 15 months after inoculation, the shortest period after inoculation being 339 days. This early occurrence of induced lung tumours as compared with those occurring spontaneously is in favour of the condensate having some carcinogenic effect. Looking at the time factor in another way the average time for cigarette condensate tumours was 533 days (76 weeks) ; for the one eucerin tumour, 609 days (87 weeks) and for spontaneous tumours 604 days (86 weeks). Thus the mean time for the occurrence of the condensate tumours was earlier than the mean for the control or for the spontaneous. When the time occurrence of the tumours according to histological type is considered, it is found that the carcinomata in the rats inoculated with the condensate occurred on an average of 506 days (72 weeks) and the spontaneous carcinoma at 556 days (79 weeks). The sarcomata in the animals inoculated with condensate were found on an average at 624 days (89 weeks), with eucerin, the control, 609 days (87 weeks) and the spontaneous sarcomata at 620 days (89 weeks). Thus the carcinomata arising in the rats inoculated with condensate occurred on an average 50 days earlier than the spontaneous. The sarcomata on the other hand were found at practically the same time in all groups. It is of interest to note that Wynder *et al.* (1953) observed that the mean time for the appearance of carcinomata in mice after the direct application of condensate to the skin was 71 weeks, that is almost at the same mean time as the carcinomata in the lungs of rats inoculated with condensate were observed in the present study. This time factor may in part be the explanation of the failure to produce tumours by other workers whose experiments did not extend for over 18 months.

In my 1957 experiments where the condensate from cigarette filters was used for inoculation, the carcinoma produced in the lung of a rat occurred at 506 days (72 weeks) and the sarcoma at 607 days (87 weeks). It thus seems obvious from all these results that tobacco condensate has to be in contact with the tissues of the lung for a long time before exciting a neoplastic response. Indeed as Graham *et al.* (1957) have pointed out cigarette condensate requires to be applied to the skin of mice or of rabbits for about half the animal's life before cancer may appear.

In the human subject bronchial carcinoma occurs with increasing frequency after 45 to 50 years of age in males, reaching its maximum between 65 and 70. This is also suggestive that if cigarette smoke is a cause of lung cancer in man it must act over a long period of time, as in the experimental animal. What possibly happens is that the oily droplets of smoke, measuring 0·3 to 1 $\mu$. in diameter, are inhaled and become deposited on the surface of the bronchial epithelium. The amount of inhaled particulate matter retained in the lungs must vary enormously depending on the condition of the bronchial mucous membrane. Ordinarily the healthy bronchial mucous membrane would, by means of ciliary action, expel most of these particles, but if the ciliated epithelium has been destroyed in places and replaced by non-ciliated, either cubical or squamous, as a result of disease or by living in a highly-polluted urban atmosphere, or as a result of habitual cigarette smoking, particles inhaled would be liable to be retained.

The ultimate fate of particles retained in the lung is of interest. As has been shown in this paper, particles of the condensate are carried away by macrophages from the site of inoculation into the lymphatics of the lung The same happens in the human subject with retained soot particles, most of which are transported in the peribronchial lymphatics towards the hilum of the lung. Any chronic peribronchial inflammation such as could arise from recurrent bronchitis would be liable to close some of these lymphatics, resulting in the arrest of the particles with the formation of focal accumulations recognisable by the naked-eye around the bronchi. If the particles have any carcinogenic power, this would be enhanced with the greater concentration. Indeed, it would be possible in the case of a smoker who inhales and who lives or had lived for a time in a highly smoke-polluted urban atmosphere to have several types of particles in the same focus of accumulation, one type exerting a co-carcinogenic effect on another with a weak carcinogenic effect. This may be the explanation of the greater incidence of bronchial carcinoma in urban areas, the retained soot particles from the polluted atmosphere acting as co-carcinogens on the particles of retained cigarette smoke which, as has been shown in this paper, appears to be a weak carcinogen.

The higher death rate from bronchial carcinoma among United Kingdom immigrants in South Africa as reported by Dean (1959) and in New Zealand by Eastcott (1956) might be explained on the above ground. In the former country, which has the highest consumption of cigarettes in the world, most of the immigrants came from the cities in the United Kingdom and were in their twenties. As a result of climatic and polluted atmospheric conditions, together with respiratory infections due to the high-density of population in these cities, these immigrants may have suffered from repeated attacks of bronchitis at an early age, with resulting focal loss of the bronchial columnar ciliated epithelium and replacement by non-ciliated. If these subjects, after they settled in South Africa or New Zealand, were heavy smokers of cigarettes, and about this neither of these authors give detailed information, then, on account of the previous change in the bronchial epithelium, particles of inhaled cigarette condensate would be retained and exert any carcinogenic effect they might possess. It is of some significance that Dean noted in his study that many of the patients, dying of lung cancer, had suffered from recurrent bronchitis.

SUMMARY

In the first series of experiments cigarette condensate prepared in a smoking machine and suspended in eucerin when inoculated into the lung of rats, guinea-pigs and rabbits after thoracotomy caused in order of time proliferation of the basal cells of the bronchial epithelium, squamous metaplasia, followed by hyperplasia, " carcinoma *in situ* " and finally squamous epithelioma. Papillomata were also produced.

In the second series of experiments a pure strain of rats was inoculated with cigarette condensate and allowed to live until death. In these 8 (11·1 per cent) malignant lung tumours were produced whereas in the controls inoculated with eucerin alone one (2·3 per cent) malignant tumour occurred and in a group of rats not inoculated into the lungs 4 (1·4 per cent) spontaneous malignant pulmonary tumours were found. In all groups most of the neoplasms occurred in animals over 18 months old. In the period up to 15 months old, only malignant tumours associated with cigarette condensate occurred.

The expenses of this work were borne by grants from the Anna Fuller Fund and from the Medical Research Council. I wish to record my thanks to the Chester Beatty Institute for the supply of rats and to Dr. H. R. Bentley of the Imperial Tobacco Company of Great Britain for some of the cigarette condensate used in the experiments. My former technician, Mr. Geoffrey Brown, helped me with all the experimental work and also prepared all the histological material. Without his loyal assistance the work would not have been possible. I am also grateful to Mr. Peter Crocker for the photographs and to Miss Mauveen Ash for secretarial assistance.

### REFERENCES

Auerbach, O., Petrick, T. G., Stout, A. P., Statsinger, A. L., Muehsam, G. E., Forman, J. B. and Gere, J. B.—(1956) *Cancer*, **9**, 76.

*Idem*, Gere, J. B., Forman, J. B., Petrick, T. G., Smolin, H. J., Muehsam, G. E., Kassouny, D. Y. and Stout, A. P.—(1957) *New Engl. J. Med.*, **256**, 97.

Blacklock, J. W. S.—(1957) *Brit. J. Cancer*, **11**, 181.

Bullock, F. D. and Curtis, M. R.—(1930) *Amer. J. Cancer*, **14**, 1.

Campbell, J. A.—(1936) *Brit. J. exp. Path.*, **17**, 146.—(1942) *Brit. med. J.*, i, 217.

Chang, S. C.—(1957) *Cancer*, **10**, 1246.

Cooper, R. L. and Lindsey, A. J.—(1955) *Brit. J. Cancer*, **9**, 304.

Curtis, M. R., Bullock, F. D. and Dunning, W. F.—(1931) *Amer. J. Cancer*, **15**, 67.

Dean, G.—(1959) *Brit. med. J.*, ii, 852.

Eastcott, D. F.—(1956) *Lancet*, i, 37.

Engelbreth-Holm, J. and Ahlmann, J.—(1957) *Acta path. microbiol. scand.*, **41**, 267.

Essenberg, J. M.—(1952) *Science*, **116**, 561.

*Idem*, Horowitz, M. and Gaffrey, E.—(1955) *West. J. Surg.*, **63**, 265.

Flory, C. M.—(1941) *Cancer Res.*, **1**, 262.

Gellhorn, A., Klausner, C. and Hibbert, J.—(1956) *Proc. Amer. Ass. Cancer Res.*, **2**, 109.

Graham, E. A., Croninger, A. B. and Wynder, E. L.—(1957) *Cancer Res.*, **17**, 1058.

Gwynn, R. H. and Salaman, M. H.—(1954) *Rep. Brit. Emp. Cancer Campgn.*, **32**, 172.

Hamer, D. and Woodhouse, D. L.—(1956) *Brit. J. Cancer*, **10**, 49.

Hamilton, J. D., Sepp, A., Brown, T. C. and Macdonald, F. W.—(1957) *Canad. med. Ass. J.*, **77**, 177.

Iles, W. G. and Sharman, C. F.—(1957) *J. appl. Chem.*, **7**, 384.

LEUCHTENBERGER, C., LEUCHTENBERGER, R. AND DOOLIN, P. F.—(1958) *Cancer*, **11**, 490.
LINDSEY, A. J.—(1959) *Brit. med. J.*, i, 506.
LORENZ, E., STEWART, H. L., DANIEL, J. H. AND NELSON, C. V.—(1943) *Cancer Res.*, **3**, 123.
MACKENZIE, I. AND ROUS, P.—(1941) *J. exp. Med.*, **73**, 391.
MAGNUS, H. A.—(1939) *J. Path. Bact.*, **49**, 21.
MÜHLBOCK, O.—(1955) *Ned. Tijdschr. Geneesk.*, **99**, 2276.
PASSEY, R. D.—(1955) *Rep. Brit. Emp. Cancer Campgn.*, **33**, 59.
*Idem*, ROE, E. M. F., MIDDLETON, F. C., BERGEL, F., EVERETT, J. L., LEWIS, G. E., MARTIN, J. B., BOYLAND, E. AND SIMS, P.—(1954) *Ibid.*, **32**, 60.
PULLINGER, B. D.—(1943) *J. Path. Bact.*, **55**, 301.
RATCLIFFE, H. L.—(1940) *Amer. J. Path.*, **16**, 237.
ROCKEY, E. E., KUSCHNER, M., KOSAK, A. I. AND MAYER, E.—(1958) *Cancer*, **11**, 466.
ROFFO, A. H.—(1937) *Dtsch. med. Wschr.*, **63**, 1267.—(1939) *Bol. Inst. Med. exp. Cáncer B. Aires*, **16**, 255.
ROGERS, J. B. AND BLUMENTHAL, H. T.—(1960) *Cancer Res.*, **20**, 191.
SCHÜRCH, O. AND WINTERSTEIN, A.—(1935) *Z. Krebsforsch.*, **42**, 76.—(1937) *Ibid.*, **46**, 414.
SUGIURA, K.—(1956) *Gann*, **47**, 243.
WYNDER, E. L., GRAHAM, E. A. AND CRONINGER, A. B.—(1953) *Cancer Res.*, **13**, 855.—(1955) *Ibid.*, **15**, 445.
WYNDER, E. L., LUPBERGER, A. AND GRENER, C.—(1956) *Brit. J. Cancer*, **10**, 507.
*Idem* AND WRIGHT, G.—(1957) *Cancer*, **10**, 255.